# Modeling the potential distribution of *Wesselsbron, Sindbis,* and *Middelburg* viruses and their vectors in Africa under future climatic and land-use changes

**DOI:** 10.1371/journal.pntd.0014072

**Published:** 2026-03-04

**Authors:** Maureen Nabatanzi, Selina L. Graff, Kigai E. E. Bigala, Peter Z. Sabakaki, Teddy A. Tindyebwa, Julius J. Lutwama, Innocent B. Rwego, Anthony M. Nsubuga, Sandra Junglen, Lisa Biber-Freudenberger

**Affiliations:** 1 Center for Development Research (ZEF), University of Bonn, Bonn, Germany; 2 Institute of Virology Charité - Universitätsmedizin Berlin, Berlin, Germany; 3 College of Veterinary Medicine, Animal Resources and Bio-Security, Makerere University, Kampala, Uganda; 4 Department of Plant Sciences, Microbiology and Biotechnology, Makerere University, Kampala, Uganda; 5 Uganda Virus Research Institute, Entebbe, Uganda; 6 Institute of Crop Science and Resource Conservation, University of Bonn, Bonn, Germany; Texas Tech University, UNITED STATES OF AMERICA

## Abstract

**Background:**

Outbreaks of zoonotic arboviruses originating in Africa occur amidst complex ecological changes and are increasingly emerging as important neglected tropical diseases. Despite sporadic epizootics and human cases of *Wesselsbron virus* (WSLV), *Sindbis virus* (SINV), and *Middelburg virus* (MIDV) in Africa, knowledge of associated risks remains insufficient for prevention.

**Methodology:**

We developed species distribution models for the three viruses alongside five key vectors, *Aedes circumluteolus and Aedes mcintoshi* for WSLV; *Culex univittatus* and *Culex pipiens* for SINV; and *Mansonia africana* and *Aedes mcintoshi* for MIDV that indicate areas with ecological suitability for the arboviruses in Africa. We integrated virus and mosquito species occurrence data with climate and land-use data for current (2015) and future (2021 – 2040) scenarios under two shared socioeconomic pathways of emission and climate projections. We applied the Maxent algorithm and evaluated over 100 candidate models per species, selecting those with above-random predictive performance based on high mean Area Under the Curve ratios (Range = 1.45 – 1.88).

**Results:**

Our models revealed high ecological suitability for the five mosquitoes in Equatorial and Southern Africa and predict emerging ecologically suitable hotspots for arboviral presence in Southern and Eastern regions, with potential future expansion into North and West Africa. Changing patterns in precipitation, especially precipitation in dry and warm seasons, urbanization, human population, livestock density, and climate change exacerbated the geographic expansion of vectors and ecological risk for arbovirus presence. While the ecological risk to arbovirus presence was currently higher in rural areas, our projections indicated a potential future shift towards urban areas.

**Conclusion:**

Our study describes how ecological changes are shaping current and future ecological risk of neglected arboviral diseases in Africa and provides spatial maps to aid intersectoral targeted surveillance and vector control as part of early-warning systems.

## Author summary

Mosquito-borne arboviral diseases are a global health concern, particularly in Africa, where rich biodiversity and tropical climates favour vector breeding and virus emergence. *Wesselsbron virus* (WSLV), *Sindbis virus* (SINV), and *Middelburg virus* (MIDV) were first detected in Africa and have the potential to cause human and animal outbreaks, yet this risk has not been mapped. Our study sought to describe the role of climatic and land-use changes in the geographical distribution of the viruses and five suspected vectors. We developed species distribution models based on the species’ known occurrence, current and future climatic and land-use conditions. We mapped areas that are ecologically suitable for vector and arbovirus presence, highlighting potential human exposure. Key predictors of suitable habitats for the vectors were precipitation during dry and warm seasons, urbanization, and human and livestock presence. Notably, ecological risk for arboviral presence may be higher in rural areas, but with changes in ecology and land use, it could increase even in urban areas. Our findings reveal pronounced spatial heterogeneity and projected shifts in vector and arbovirus presence given ecological changes. In settings where the evidence for vector-host-virus ecology is underdeveloped, our ecological risk maps can indicate areas that could be targeted for vector control and intersectoral surveillance to strengthen early warning.

## Introduction

Arthropod-borne infections account for almost half of the recent emerging infectious diseases, particularly in countries of the global south [1]. *Wesselsbron virus* (WSLV), belonging to the family Flaviviridae and genus *Flavivirus, was* first identified in 1955 in South Africa [2]. Human disease is characterized by fever, headache, arthralgia, and myalgia [3]. The reported geographical distribution is primarily in Africa and Thailand [4], with sporadic human cases and antibodies to WSLV detected in South Africa, Uganda, Nigeria, Mauritania, and Senegal [3,5–8]. WSLV has been detected from multiple mosquito taxa, including several *Aedes (Ae)*, *Anopheles*, *Culex (Cx)*, and *Mansonia* (*Ma*) species, but its main vectors are freshwater breeding *Aedes* species, notably, *Ae. circumluteolus*, *Ae. mcintoshi* and *Ae. caballus* [4,9,10]. In Africa, WSLV poses a substantial zoonotic threat to humans, exemplified by its broad geographical circulation in mosquitoes, amplification in multiple vertebrates such as rodents, other wild animals, and epizootic potential in livestock [5,11–13]. Domestic sheep are highly susceptible (abortions, neonatal mortality), with cattle and goats also acting as amplifying hosts, while humans are incidental hosts [9,14].

*Sindbis virus* (SINV), an alphavirus (family *Togaviridae) which was* first isolated in Egypt in 1952, is widely distributed across Africa, Europe, the Middle East, and parts of Asia [15–17]. In humans, SINV infections are characterized by febrile illness, widespread maculopapular rash, myalgia, and prolonged arthralgia [18,19]. In northern Europe, SINV is endemic in Sweden and Finland, causing thousands of symptomatic human cases during peak years; persistent low-level circulation is linked to resident bird populations and *Culex* vectors [19,20]. In South Africa, epidemics associated with *Cx. univittatus* have been documented for decades, with large outbreaks recorded in the 1970s – 80s affecting thousands of humans and sporadic activity continuing thereafter [21,22]. Sporadic human cases were reported in Uganda, China, Australia, and Spain [15,18,23]. Human infections arise from bites by infectious mosquitoes, an effect of spill-overs from enzootic circulation in birds [24]. Transmission events are dominated by multiple ornithophilic *Culex* mosquitoes, notably, *Cx. univittattus* and *Cx. pipiens* in Africa and *Cx. torrentium/pipiens* in Europe where *Ae. cinereus* is a bridge vector [4,18,22,24,25]. The enzootic cycle relies on avian amplifying hosts such as thrushes (*Turdus spp.*) and crows (*Corvus corone sardonius*), implicated as reservoirs [18,24,26]. Cross-species spill-over is likely enhanced by a broad vertebrate host range, including multiple wild and domestic animals like horses [15,16,27].

*Middelburg virus* (MIDV) of the family *Togaviridae*, genus *Alphavirus* was first isolated in South Africa in 1957. The virus is thought to circulate primarily in enzootic transmission cycles involving mosquitoes and vertebrate hosts, with episodic spill-over into humans and domestic animals [28–30]. In a South African study, Fourie et al (2022) suggested that humans infected with MIDV disease could present with fever and neurologic symptoms [31]. Overall, formal vector competence and vertical transmission studies for MIDV remain scarce. Recent entomological investigations in South Africa detected MIDV in several mosquito genera, including *Aedes* and *Culex*, supporting the likelihood of a multi-vector system [32]. In Uganda, MIDV was detected in *Mansonia africana* and *Eretmapodites intermedius*, suggesting possible enzootic transmission in forested ecosystems [33]. Floodwater *Aedes* species, particularly *Aedes mcintoshi*, have been repeatedly highlighted as ecologically important due to their association with rainfall-driven emergence, potential involvement in vertical transmission, and frequent detection in arbovirus surveillance studies [9,28]. Serological and virologic evidence indicate MIDV exposure in a range of wild and domestic animals, including livestock, implicating mammals as amplifying hosts during periods of increased vector abundance [27]. MIDV has demonstrated epizootic potential, especially among sheep, and has been associated with severe neurological disease in horses, livestock, and wildlife, indicating its pathogenicity in mammals [27,29,34].

Anthropogenic environmental change and climate variability increasingly influence the epidemiology of arboviral diseases by reshaping the distribution of mosquito vectors, vertebrate hosts, and transmission dynamics [35,36]. Land-use change, urbanization, invasion of forests, and socioeconomic activities affect vegetation cover, mosquito density, and biting behaviour, increasing contact among wildlife, livestock, and humans [36,37]. At the same time, globalization has facilitated the spread of mosquitoes beyond their historical ranges, accompanied by ecological and behavioural adaptations that enable persistence in diverse urban and peri-urban environments [38].

These processes are well documented for *Ae. aegypti* and *Ae. albopictus*, whose urban adaptation has driven the global expansion of Dengue, Zika, and Chikungunya viruses [39,40]. In contrast, many arboviruses of African origin remain poorly characterized despite evidence that Africa’s diverse ecosystems host numerous competent or suspected vectors [8,41–43]. Rapid environmental change may further alter vector distributions and transmission pathways, increasing the potential for emergence within and beyond Africa [40]. *Wesselsbron virus* (WSLV), *Middelburg virus* (MIDV), and *Sindbis virus* (SINV) were first described in Africa between the 1950s and 1970s, yet their contemporary epidemiology and epidemic potential remain poorly understood [2,4,7,11,23,29]. Although detected in sub-Saharan and East Africa, recognized outbreaks have been largely confined to Southern Africa (WSLV and MIDV) and Europe (SINV), and their potential distribution across Africa remains uncertain [9,12,15,29,31,33,44]. Surveillance gaps, exclusion from neglected tropical disease monitoring, and limited differential diagnostics further hinder understanding of these viruses, leading to underdetection or misclassification. For example, sporadic detections of WSLV were made during Rift Valley Fever (RVF) outbreaks [3,5,12]. In addition, human cases may also be misdiagnosed due to similar symptom presentation with other arboviruses and limited capacities for differential diagnosis [31]. 

While spatial modeling has effectively guided surveillance and prevention for Dengue, Zika, and Yellow fever [45–47], comparable continental-scale assessments are limited for WSLV, SINV, and MIDV.

We modeled the environmental suitability of WSLV, SINV, and MIDV and their mosquito vectors across Africa under current and future climatic and land-use conditions. By focusing on ecological suitability rather than realized transmission, this study provides a macroecological baseline to identify areas of potential virus presence and human exposure, supporting targeted surveillance and future ecological and epidemiological investigations [47,48].

## Materials and methods

### Ethics statement

This study received ethical clearance from the University of Bonn Center for Development Research and the Charité Universitätsmedizin Berlin. No human subjects were involved in this study. We used openly accessible sources for secondary data, which had no person identifiable data, and we acknowledge the authors/sources.

### Study site

Africa is the main source of mosquito-borne viruses of global public health importance [4]. In this study, we combined data from different sources across Africa, including online databases, literature, surveys, and primary data from Uganda, with ecological data covering Africa, to model the habitat suitability of mosquito and arbovirus species under current and future climatic conditions.

## Species and presence records

### Viruses

**Fig 1:**
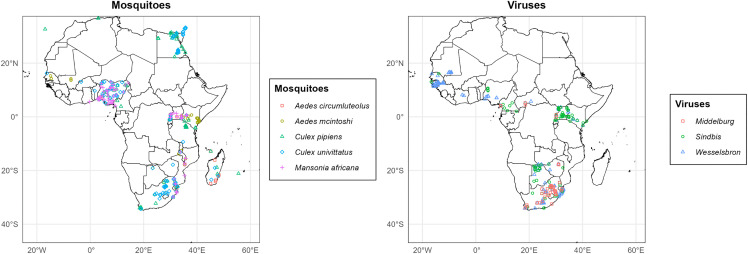
Presence points of the mosquito and virus species used in species distribution modeling; S2 Table shows the sources of the presence points. Map was plotted in R on a base layer derived from the openAFRICA “Africa Shapefiles” dataset: https://open.africa/dataset/africa-shapefiles/resource/dcdadd25-0137-4c93-ae5a-82b39d424d60, released under a Creative Commons 4.0 International License.

This study assesses the habitat suitability for three neglected arboviruses, *Wesselsbron, Sindbis*, and *Middelburg viruses*. We selected these viruses due to their epidemic potential and the insufficient attention in Africa given to potential human exposure amidst ecological change. We obtained presence records for WSLV, SINV, and MIDV. During February – March 2024, we searched the National Center for Biotechnology Information (NCBI) Virus database for all published nucleotide sequences using the following taxonomic identifications: *Wesselsbron virus* (164416), *Sindbis virus* (11034), and *Middelburg virus* (11023) [49]. We also searched NCBI’s PubMed, a literature database using the same scientific names, for studies in Africa that identified these viruses. To limit the risk of misclassification, we selected studies where one of the samples underwent virus isolation (for older studies) and polymerase chain reaction (PCR) or genome sequencing (for recent studies). We extracted the date, location, and host of the sample collection. Where location coordinates were missing, we used the sampling site description to identify proximal coordinates in Google Maps [50]. We removed duplicate records and coordinates resulting in: WSLV (n = 154), SINV (n = 109), and MIDV (n = 49) (Fig 1). S2 Table in the Supporting Information shows the sources of the presence points.

### Mosquitoes

During the literature review conducted in February–March 2024, we collated the main vector species implicated in virus epidemiology, ranking them by evidence and viral detections (S1 Table). However, the final selection of the vectors to use as predictors was constrained to those that fulfilled the former criteria but also had robust presence points in Africa sufficient to generate vector niche models that could be applied in the virus models. In Africa, WSLV has been detected in multiple *Aedes* species including *Ae. caballus*, *Ae. juppi*, *Ae. circumluteolus, Ae. mcintoshi*, *Ae. luridus*, *Ae. vexans*, *Ae. unidentatus*, *Ae. gibbinsi* and *Ae. tricholabis* as well as *Ma. uniformis*, *An. coustani* and *Culex* species. [4,8–10]. Of the 154 WSLV detections we identified, 44 were from mosquitoes, with at least 12 different species. *Ae. vexans* (7) had the highest detections, followed by *Ae. circumluteolus* (6) and *Ae. mcintoshi* (4) (S1 Table). Vector modeling was restricted to *Ae. circumluteolus* and *Ae. mcintoshi*, which are consistently identified as the primary enzootic vectors and as the epizootic vectors associated with livestock outbreaks and floodwater-driven transmission across Africa [3,9,13,51].

Multiple *Culex* species (e.g., *Cx. pipiens*, *Cx. torrentium*, *Cx. univittatus*, *Cx. perexiguus*, *Cx. quinquefasciatus, Cx. modestus*), as well as *Culiseta morsistans* and, less consistently, certain *Aedes* and *Anopheles* taxa have been reported infected with or competent for SINV [17,24,25,52]. Although many *Culex* species are implicated as vectors, SINV isolations are dominated by *Cx. univittatus* and *Cx. pipiens* in Africa, while in Europe *Cx. torrentium/Cx. pipiens, Cx. perexiguus, Culiseta morsistans*, *Ae. cinereus* and *Ae. Ochlerotatus caballus* play important roles [17,24,53,54]. Of the 109 SINV presence points we identified, 44 were from at least 17 different mosquito hosts; these were dominated by the *Culex* genus (31), featuring multiple species, led by *Cx. univittatus* (15) and *Cx. pipiens* (4) (S1 Table). Therefore, we selected *Cx. univittatus* and *Cx. pipiens* as focal vectors.

Field and molecular studies have detected MIDV in multiple *Aedes* species, including *Ae. caballus*, *Ae. mcintonshi*, *Ae. circumluteolus*, *Ae. dalzieli*, and members of the *Aedes (Aedimorphus)* group, as well as other mosquito taxa [29,30,32,33,55]. Earlier entomological investigations in southern Africa suggested that floodwater *Aedes* species, including *Aedes (Ochlerotatus) juppi*, *Ae. caballus*, and *Ae. mcintoshi*, may contribute to MIDV maintenance and episodic amplification, potentially through vertical transmission and co-circulation with other arboviruses such as WSLV and SINV [9,28]. Given this evidence and the absence of consistent competence data across species, we adopted a conservative modeling strategy. Among *Ae.* mosquitoes, we prioritized *Ae. mcintoshi* due to its ecological association with floodwater-driven transmission systems, repeated detection in recent surveillance studies, and sufficient availability of occurrence data for spatial modeling [32,55] (S1 Table). We complemented this with *Ma. africana* to represent a non-*Aedes* mosquito potentially involved in enzootic transmission [4,33].

#### Presence records for mosquitoes.

We searched the Global Biodiversity Information Facility (GBIF) database using the following GBIF taxon identifications: *Ae. circumluteolus* (Theobald, 1908) *(1651224), Ae. mcintoshi* Huang, 1985 (1651403), *Cx. univittatus* Theobald, 1901 (1653264), *Cx. pipiens* Linnaeus, 1758 (1652991), *Cx. perexiguus* Theobald, 1903 (1653140), *Ma. africana* (Theobald, 1901) (5089851) [56]. Additional presence points were obtained from mosquito sampling in Uganda [33]. The extracted data were cleaned to remove duplicates resulting in *Ae. circumluteolus* (n = 121), *Ae. mcintoshi* (n = 89), *Cx. univittatus* (n = 172), *Cx. pipiens* (n = 241) and *Ma. africana* (n = 157) (Fig 1).

### Ecological variables

This study integrated climatic, environmental, and land-use data to represent the ecologies of mosquitoes and viruses which contribute to disease transmission [57]. We selected precipitation, temperature, forest cover, normalized difference vegetation index, NDVI (NDVI), agriculture, human population, settlements, and urbanization as key predictors. These variables have been found to influence mosquito survival, breeding, and virus transmission [47,48,58]. Where available, we chose temporal scales of 2015 (current) and 2040 (future) to align species presence records with available environmental data [59].

#### Climatic variables.

We used WorldClim version 2.1, whose data are derived from over 9,000 weather stations, to obtain 19 bioclimatic variables for 1970–2000; the variables represent key ecological processes [60] (S3 Table). Shared Socioeconomic Pathways (SSPs) are the Intergovernmental Panel on Climate Change’s projections of future emissions and climate. We chose SSP2-4.5 for moderate and SSP5-8.5 for severe conditions [61]. We selected IPSL - CM6A - LR and HadGEM - GC31 - LL Global Climate Models (GCM) from the Coupled Model Intercomparison Project Phase 6 for their strong performance in Africa [62,63]. We downloaded data for 2021–2040 for these models and SSPs from WorldClim [60].

#### Livestock.

We accessed livestock data from the Food and Agriculture Organisation’s Gridded Livestock of the World (GLW) version 4 for 2015 [64]. Since high-resolution livestock datasets for future scenarios are not available, we used the GLW data version 4 from 2020, a high-resolution compilation of livestock densities [65], to model future suitability and assumed no change in densities.

#### Human population and urbanization.

We extracted human population and settlement model grids (urbanization) for 2015 and built-up areas for 2014 from the “Global Human Settlement Layer” dataset [66,67]. For future human populations, we accessed projections for SSP2-4.5 and SSP5-8.5 [68].

#### Cropland.

We extracted the Global Land Analysis and Discovery laboratory’s data for 2012–2015, comprising the percent of croplands per pixel [69]. We derived future land-use from the Global Change Analysis Model and Demeter datasets for SSP2-4.5 and SSP5-8.5 for 2040 at 0.05-degree resolution and extracted layers for crops, forests and urban land - use [70].

#### Forest.

We downloaded the Moderate Resolution Imaging Spectroradiometer (MODIS) Land Cover Type (MCD12Q1) Version 6.1 dataset of the International Geosphere-Biosphere Programme for 2015 and extracted forested areas [71,72]. To estimate future forested areas in 2040, we utilized the Global Change Analysis Model and Demeter datasets for SSP2-4.5 and SSP5-8.5 [70].

#### Normalized difference vegetation index.

We extracted the NDVI data for 2015 at 1 kilometer (km) resolution from the Terra MODIS Vegetation Indices (MOD13A3) Version 6.1 monthly dataset [72,73]. For future scenarios, high-resolution NDVI data for Africa was unavailable.

### Suitability modeling

The ecological niche of vectors, hosts, or reservoirs indicates geographical areas with potential for virus transmission [74]. We applied the suitability distribution models of vectors as predictor variables in the models of the respective viruses.

#### Pre-modeling data processing.

The final predictor dataset comprised 26 environmental variables (Table 1); for detailed descriptions, see S3 Table). We processed all variables to cover a uniform spatial extent (Africa), with the same projection (EPSG: 4326-WGS 84), resolution (0.00833 × 0.00833 grid cell size where 1-degree latitude ~111.32 km^2^ per grid), alignment, and ASCII grid format [75]. We used R version 4.4.1 and the packages “sf” and “terra” to process the data [76–78].

**Table 1:** Ecological variables used in the study.

**Variables (n = 26)**
19 Bioclimatic variables: Bio1 = Annual Mean Temperature; Bio2 = Mean Diurnal Range (Mean of monthly (max temp - min temp)); Bio3 = Isothermality (Bio2/Bio7) (×100); Bio4 = Temperature Seasonality (standard deviation ×100); Bio5 = Max Temperature of Warmest Month; Bio6 = Min Temperature of Coldest Month; Bio7 = Temperature Annual Range (Bio5-Bio6); Bio8 = Mean Temperature of Wettest Quarter; Bio9 = Mean Temperature of Driest Quarter; Bio10 = Mean Temperature of Warmest Quarter; Bio11 = Mean Temperature of Coldest Quarter; Bio12 = Annual Precipitation; Bio13 = Precipitation of Wettest Month; Bio14 = Precipitation of Driest Month; Bio15 = Precipitation Seasonality (Coefficient of Variation); Bio16 = Precipitation of Wettest Quarter; Bio17 = Precipitation of Driest Quarter; Bio18 = Precipitation of Warmest Quarter; Bio19 = Precipitation of Coldest Quarter
Normalized Difference Vegetation Index
Human population
Built-Up areas
Settlement model grid
Forested areas
Livestock density
Croplands

#### Species distribution modeling.

Species distribution models (SDMs) use ecological variables and presence points of species to predict the habitat suitability of a species across the landscape. They indicate where conditions are suitable for the species on a continuous scale (0–1). SDMs are widely applied in studying the current and future habitat suitability of vectors and diseases under climatic changes [46,47,58,79]. Maximum entropy (Maxent), a popular SDM method, predicts habitat suitability based on species presence and environmental conditions and projects to future environmental scenarios. The resulting species distribution maps indicate where conditions are suitable for the species on a continuous scale (0–1) [59,80]. Presence maps show where the species are estimated to occur based on thresholds applied to the suitability models. They convert suitability into stacked levels of certainty of species presence, indicating where the species are likely present with higher confidence. Species distribution models that use presence-only species data are prone to spatial bias that can be introduced by differing methods and intensity of species sampling across studies [59]. We utilized the target-group background approach to minimize sampling bias by using collective occurrence records of the genera to which our study species belong to reflect the general sampling effort [81,82]. Therefore, occurrences in Africa of the genera *Aedes* Meigen, 1818*(*for species *Ae. circumluteolus* and *Ae. mcintoshi)*, *Culex* Linnaeus*,* 1758 (for species *Cx. univittatus* and *Cx. pipiens),* and *Mansonia* Blanchard*, 1901 (*for species *Ma. africana)* were extracted from GBIF (S2 Table). We converted the genera’s sampling points into gridded layers using kernel density estimations under the R package “MASS” and incorporated these in the respective Maxent models of the vectors [81,82].

In the first step, we ran Maxent version 3.4.4 [59,80], using all ecological variables, a genus-specific bias grid, automatic feature classes, and split the occurrence data into 75% training and 25% testing. We used the jackknife test, set the regularization multiplier to 3, conducted 5 cross-validation runs, set a convergence threshold of 1.0e-5, used a maximum of 10,000 background points and 500 maximum iterations. In a second step, we considered the averages of the cross-validation runs to drop variables of low importance and high Pearson correlation (over 0.7) [75]. See the correlation matrix in S1 Fig. This enabled the exclusion of less important predictors and addressed multicollinearity in subsequent species modeling.

#### Parameter setting optimization.

Selection of an appropriate combination of settings in Maxent is challenging. In the third step, we used the “kuenm” approach to optimize the calibration of multiple candidate models, selection, evaluation, and projection [83]. We organized occurrence data into four sets: random independent points (10), joint (minus independent points), training (70% of joint), and testing (30% of joint). Per species, we combined the variables chosen in step 2 with one of two regularization multipliers (3 or 5) and a basic combination of feature classes to produce sets of variables and candidate models. During each calibration, we created a candidate model using each joint and training set of occurrences.

#### Candidate model evaluation.

We measured statistical significance using the partial receiver operating characteristic (pROC), 50% of data for bootstrapping, and 500 iterations [84]. We assessed predictive ability by the omission rate (OR) and allowed a 5% omission error (E) [85]. We evaluated model complexity using the Akaike Information Criterion corrected (AICc) for small sample sizes [83]. We selected best-performing candidate models based on statistical significance, OR below 5%, and delta AICc less than 2. S4 Table shows the model settings and results.

#### Final models.

We generated final models using the best - performing settings with five bootstrap replicates, projecting them with extrapolation and clamping based on future GCM and SSP variables. Each model was evaluated using independent points (S4 Table). Output models presented as maps show predicted habitat suitability probabilities ranging from 0 (least suitable) to 1 (most suitable). We calculated suitability differences between current and future models. For mosquitoes, we reclassified suitability into low (0 - 0.3), average (0.4 - 0.6), and high (0.7 - 1) categories, estimating the area (km^2^) per class. We converted suitability models into species presence models by assigning presence to cells with suitability above four thresholds: 1) Equal training sensitivity and specificity; 2) Maximum training sensitivity plus specificity; 3) Balanced training omission; and 4) Ten-percentile training presence. This created a stack of four presence/absence models per species and scenario, showing the number of threshold criteria (0–4) under which a given cell is predicted as suitable. For viruses, we combined these stacks to produce hotspot maps highlighting areas with the highest certainty of species presence (value 12) and least certainty (value 0). We overlaid hotspot maps with human population data for 2015 and 2040 to visualize population as a predictor of virus presence and likely exposure. We used R Studio and the packages “ggplot2”, “terra”, “viridis”, and “gridExtra” to visualize figures [76,78,86–88]. Administrative boundary data were obtained from the openAFRICA “Africa Shapefiles” dataset released under a Creative Commons 4.0 International License.

## Results

We modeled habitat suitability for three viruses (WSLV, SINV, and MIDV) and five mosquito vectors (*Ae. circumluteolus, Ae. mcintoshi*, *Cx. univittatus*, *Cx. pipiens* and *Ma. africana)*. We analyzed at least 100 candidate models for each mosquito or virus species, most of which were significantly better in predicting species presence than a randomized prediction (S4 Table). The best candidate models had above-random habitat suitability predictions with high mean Area Under the Curve (AUC) ratios (Range = 1.45 - 1.88). Final models produced using the best candidate model settings resulted in higher mean AUC ratios (Range = 1.67 - 1.97) implying improved fitness of training data and better predictive performance (S4 Table). To facilitate interpretation, habitat suitability outputs (continuous probabilities from 0 to 1) were converted into threshold-based presence maps summarizing agreement across four presence–absence criteria. The resulting presence index (0–4) reflects increasing certainty of environmentally suitable conditions for virus presence.

### Habitat suitability for vectors

Under current ecological conditions, we predicted varied suitability for the five mosquitoes, particularly across Equatorial and Southern African countries. Suitability was predicted to remain relatively stable under both scenarios during 2021–2040 with small geographical expansions for *Cx. univittatus, Ma. africana, Ae. mcintoshi,* and *Cx. pipiens*. Areas with high precipitation in warm (1,300mm) and cold quarters (3,000mm) were suitable for *Ae. cicumluteolus* but not *Ae. mcintoshi*. Both *Aedes* species required at least 23°C for average to high suitability and preferred temperatures less than 34°C, while *Culex* species’ suitability was sensitive to extremely low (below 7°C) and high temperatures (above 32°C). For *Aedes* and *Culex* species, we found average suitability values in areas that received at least 400 mm of precipitation in dry quarters. Suitability was positively influenced by built-up areas (for *Culex* species) and livestock (for all mosquitoes, except *Ma. africana*).

**Fig 2:**
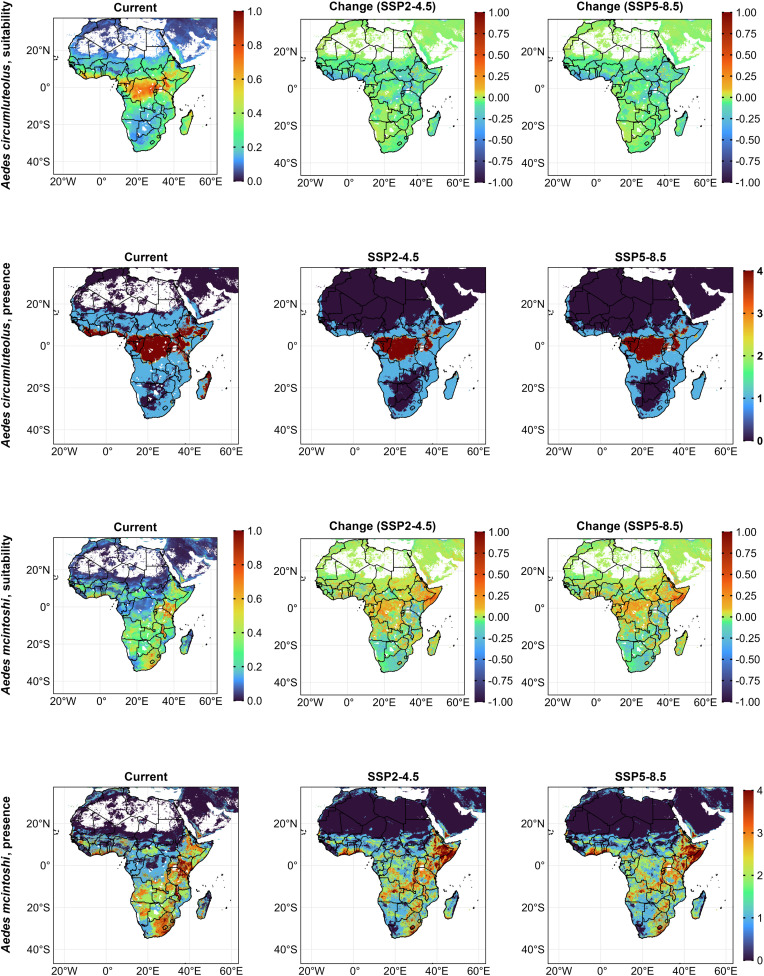
Habitat suitability and presence for *Ae. circumlutelous* and *Ae. mcintoshi* currently and under future (2021 – 2040, SSP2-4.5 and SSP5-8.5) ecology. For suitability scenarios, positive values indicate an increase, and negative values, a decrease in suitability. Presence maps display a threshold-based certainty index (from least certainty (0) to highest certainty (4)) representing the number of presence–absence models in which suitability exceeded predefined thresholds. Africa base layer derived from the openAFRICA “Africa Shapefiles” dataset: https://open.africa/dataset/africa-shapefiles/resource/dcdadd25-0137-4c93-ae5a-82b39d424d60, released under a Creative Commons 4.0 International License.

We predicted areas with highly suitable habitats for *Ae. circumluteolus* primarily in equatorial countries from the West African coast to Central and Eastern Africa, including the North-Eastern horn (Fig 2). Currently, areas with low suitable habitats cover 2,360,000 km^2,^ increasing under SSP2-4.5 (42%) and SSP5-8.5 (43%). In contrast, highly suitable habitats were limited to 176,000 km^2^, declining under SSP2-4.5 (-49%) and SSP5-8.5 (-53%) (Fig 5 and S5 Table). Current highly suitable habitats were concentrated in Central and Eastern Africa and Madagascar. Future projections showed the highest certainty of presence was limited to Central and Eastern Africa (Fig 2). The key predictive variables were isothermality (47%) and precipitation of the driest month (35%). The least important were built-up areas (0.12%) and forests (0.03%). Suitability increased with isothermality, minimum temperature (from 0.2 at -30°C to 0.5 at 23°C), precipitation of the driest month (from 0.6 at 8mm to 0.8 at 140mm), warmest quarter precipitation (0.5 at 200mm to 0.9 at 1,300mm), coldest quarter precipitation (steady at 0.5 from 1mm to 3,500mm), and livestock (from 0.7 at 0 animals to 0.95 at 550,000 animals per 111km^2^).

We found areas with high suitability for *Ae. mcintoshi* mainly in East Africa, Malawi, and Southern Africa, with increased suitability across Sub-Saharan Africa (SSA), particularly in the Democratic Republic of Congo (DRC), Ethiopia, and Somalia under SSP2–45 and SSP5–85 (Fig 2). Areas with low suitability covered the most area (2,346,000 km^2^) and were expected to increase by 32%, while highly suitable habitats (101,000 km^2^) were projected to decline under SSP2-4.5 (-31%) and SSP5-8.5 (-34%) (Fig 5, and S5 Table). Future presence was predicted to spread to Central Africa, Ethiopia, and Somalia (Fig 2). Precipitation of the warmest quarter (34%) and human population (33%) were the best predictors for suitability, while livestock (1.5%) and built-up areas (0.5%), the least. Suitability responded positively to the minimum temperature of the coldest month (from 0.4 at 20 °C - 0.95 at 23 °C), precipitation of the warmest quarter (peaked at 0.6 at 100mm), built-up areas, and livestock. It responded negatively to the maximum temperature of the warmest month (plateaued at 0.55 until 34 °C, then declined), precipitation of the coldest quarter (peaks at 0.5 at 300mm, then declined to 0 at 3,000mm), forest cover, and human population.

**Fig 3:**
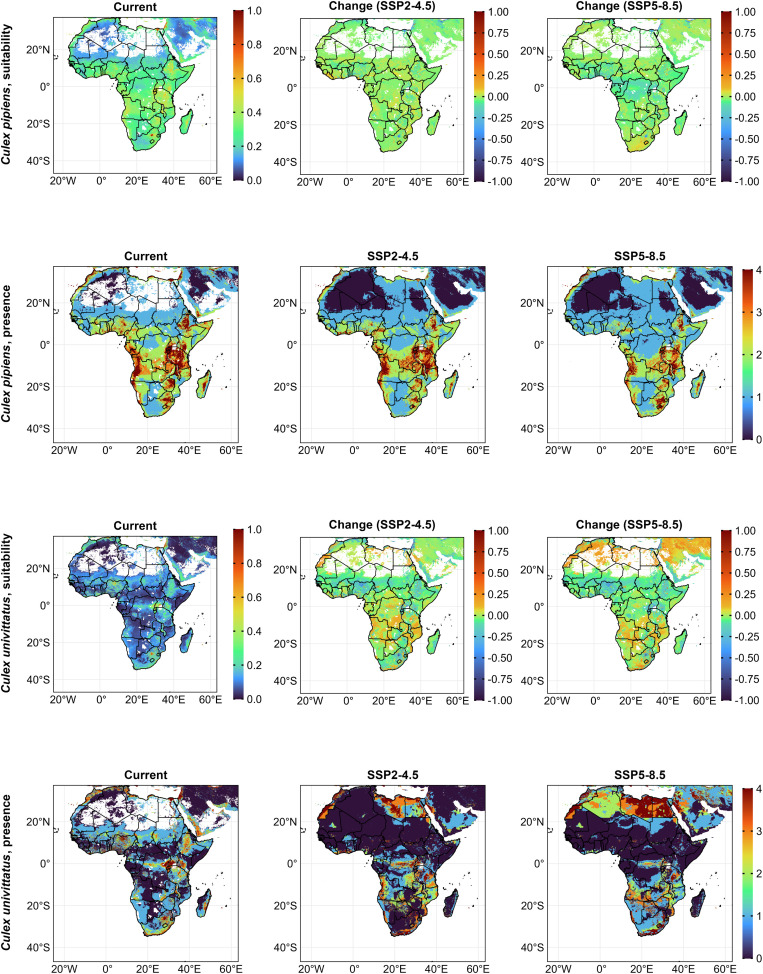
Habitat suitability and presence for *Cx. pipiens* and *Cx. univittatus* currently and under future (2021 – 2040, SSP2-4.5 and SSP5-8.5) ecology. For suitability scenarios, positive values indicate an increase, and negative values, a decrease in suitability. Presence maps display a threshold-based certainty index (from least certainty (0) to highest certainty (4)) representing the number of presence–absence models in which suitability exceeded predefined thresholds. Africa base layer derived from the openAFRICA “Africa Shapefiles” dataset: https://open.africa/dataset/africa-shapefiles/resource/dcdadd25-0137-4c93-ae5a-82b39d424d60, released under a Creative Commons 4.0 International License.

The *Cx. pipiens* model predicted low suitability across most areas (21,720,000 km^2^) with average suitability spanning 9,100,000 km^2^. High-suitability zones covered approximately 250,000 km^2^ in select regions of West Africa, the Nile Delta, eastern Africa, Angola, South Africa, and Madagascar (Figs 3 and 5). Under SSP2-4.5 and SSP5-8.5, low suitability areas increased by 39% and 46%, while highly suitable Areas declined by 75% (S5 Table). We observed the highest presence in East Africa, including Malawi, Zambia, Angola, Namibia, South Africa, and Madagascar. Built-up areas (54%) were the primary contributors to habitat suitability, followed by livestock (13%), cropland (2.6%), while precipitation in the driest quarter (1.1%) was the least significant. Suitability increased with the precipitation of the driest quarter (rising from 0.25 at 0 mm to 0.64 at 420 mm), livestock (plateauing at 0.95 with 600,000 animals per 111 km^2^), built-up areas, and human population. Conversely, suitability declined with the maximum temperature of the warmest month (starting at 0.9 from 0 °C to 15 °C, then dropping to 0.3 at 45 °C), the annual temperature range (declining from 0.6 at 8 °C to 0.1 at 46 °C), and cropland. Current habitat suitability for *Cx. univittatus* was rather low in most areas (29,370,000 km^2^) (S5 Table). High-suitability areas (310,000 km^2^) lay along the Sahara coastline, Nile Delta, South-Western Uganda, Rwanda, South Africa and Reunion Island (Fig 3). Under SSP2-4.5, areas with low suitability increased by 25% and average suitability by 13%. Under SSP5-8.5, highly suitable areas increased by 106%, particularly in the Sahara and Central/Southern Africa (Figs 3 and 5). Current high certainty of presence includes Western Africa, the Nile Delta, and parts of East and Southern Africa. Future projections under SSP2-4.5 show spread into Morocco, Libya, Egypt, and parts of Central Africa. SSP5-8.5 further expands this to Libya and Egypt (Fig 3). The best predictors for suitability are built-up areas (58%) and temperature annual range (20%), while croplands (2.5%) and forests (1%) are the lowest. Suitability increased with building footprint, and livestock (peaking at 0.9 at 1 million animals per 111 km^2^) but declined with forest and cropland covers. Negative responses were noted for minimum temperature of the coldest month (peaked at 0.6 at 7^o^C then declined), temperature annual range (from 0.3 at 14^o^C - 0.6 at 32^o^C, then declined), annual precipitation (from 0.9 at 0 mm - 0.4 at 1,500mm, then declined), and precipitation of driest quarter (peaked at 0.5 at 450mm).

**Fig 4:**
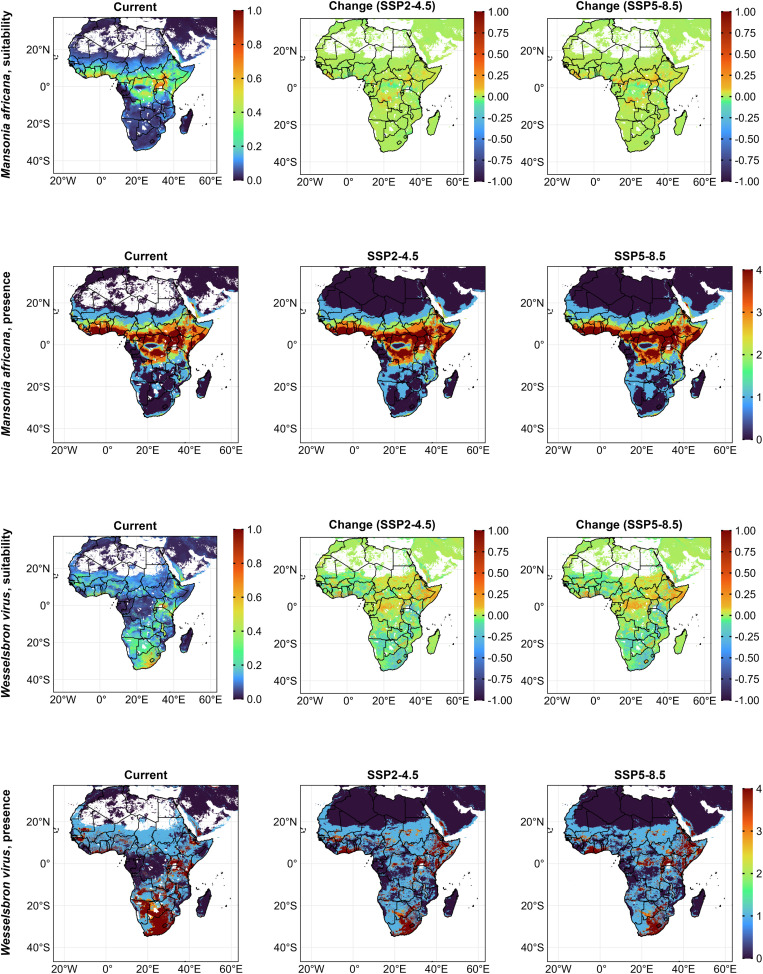
Habitat suitability and presence for *Ma. africana* and WSLV currently and under future (2021 – 2040, SSP2-4.5 and SSP5-8.5) ecology. For suitability scenarios, positive values indicate an increase, and negative values, a decrease in suitability. Presence maps display a threshold-based certainty index (from least certainty (0) to highest certainty (4)) representing the number of presence–absence models in which suitability exceeded predefined thresholds. Africa base layer derived from the openAFRICA “Africa Shapefiles” dataset: https://open.africa/dataset/africa-shapefiles/resource/dcdadd25-0137-4c93-ae5a-82b39d424d60, released under a Creative Commons 4.0 International License.

**Fig 5:**
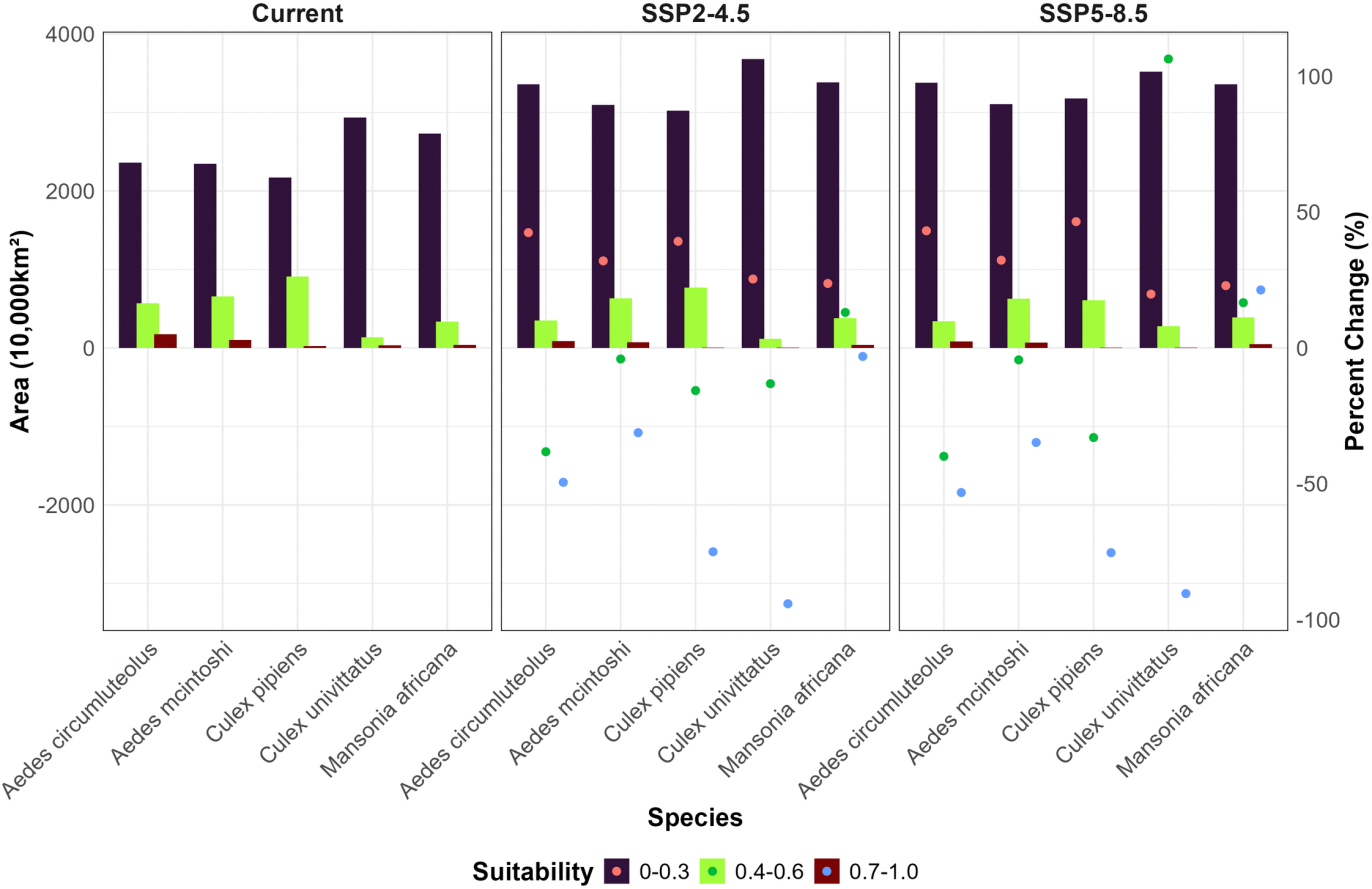
Predicted area for habitat suitability in 10,000 km^2^ per mosquito species and climate change scenario. Habitat suitability is classified into, low (0 - 0.3), average (0.4 - 0.6) and high (0.7 - 1.0). On the right y-axis, the percent change in the area of habitat suitability for future scenarios is denoted by points.

Most of Africa (27.33 million km^2^) had low suitability for *Ma. africana* with highly suitable areas around Uganda, South Sudan, Ethiopia, and Kenya (400,000 km^2^). Average suitability spanned 3.34 million km^2^ in Equatorial Africa. Under both scenarios, suitability increased around Sierra Leone, Liberia, and Central Africa. Under SSP2-4.5, low and average suitability areas increased by 13%, while high suitability decreased by 3%. Under SSP5-8.5, low suitability increased by 22%, average by 16%, and high by 21% (Figs 4, 5 and S5 Table). High certainty of presence was stable across equatorial countries in both future scenarios (Fig 4). Key predictors were isothermality (46%) and human population (24%), while mean temperature of the warmest quarter (0.034%) and precipitation seasonality (4.08%) had minimal influence. Suitability responded positively to isothermality, mean temperature of the warmest quarter (peaked at 0.6 at 17^o^C, declined to 0.15 at 37^o^C), mean temperature of the coldest quarter (peaked at 0.75 at 29^o^C), precipitation of the warmest quarter (peaked at 0.65 at 300mm then declined to 0 at 700mm), precipitation of driest quarter (peaked at 0.75 at 150mm but declined to 0 at 400mm). Suitability decreased with precipitation seasonality, human population (beyond 15,000 people per 111 km^2^), and livestock (beyond 200,000 animals per 111 km^2^).

### Predicted ecological suitability for virus presence

Current ecological suitability for WSLV was highest in South Africa, Lesotho, and East Africa. Future habitat suitability was predicted to increase in Lesotho and the equatorial countries from the west to the east of Africa. Current ecological suitability for WSLV presence was highly certain in areas of southern and eastern Africa, increasing in Ivory Coast, Ghana, Nigeria, and the Horn of Africa while declining in South Africa under future scenarios (Fig 4). Key predictors were built-up areas (33%), and habitat suitability of *Ae. mcintoshi* (25%) and *Ae. circumluteolus* (11%), with minimal contributors from livestock (0.46%) and cropland (0.81%).

**Fig 6:**
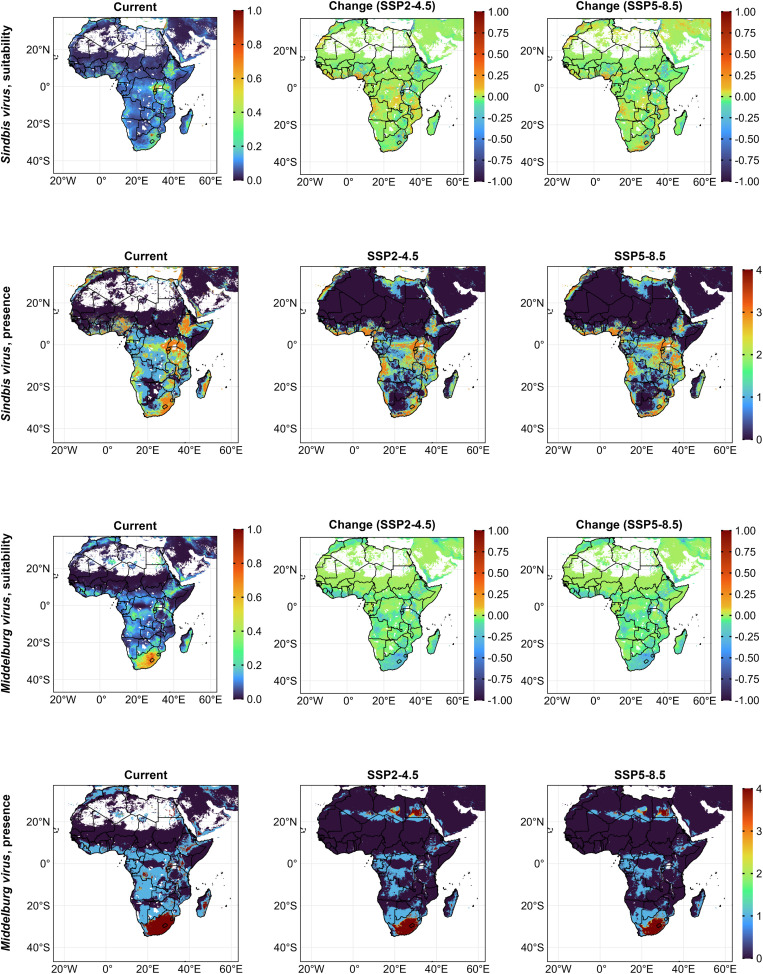
Habitat suitability and presence for SINV and MIDV currently and under future (2021 – 2040) SSP2-4.5 and SSP5-8.5 ecology. For suitability scenarios, positive values indicate an increase, and negative values, a decrease in suitability. Presence maps display a threshold-based certainty index (from least certainty (0) to highest certainty (4)) representing the number of presence–absence models in which suitability exceeded predefined thresholds. Africa base layer derived from the openAFRICA “Africa Shapefiles” dataset: https://open.africa/dataset/africa-shapefiles/resource/dcdadd25-0137-4c93-ae5a-82b39d424d60, released under a Creative Commons 4.0 International License.

Under the current ecological conditions, limited areas had above-average suitability for SINV, primarily in Nigeria, central Ethiopia, eastern Africa, in South Africa and central Madagascar. Under the future scenario SSP2-4.5, predicted ecologically suitable areas slightly increased across Africa, particularly in coastal West Africa and in countries below the equator; the increases were more obvious under SSP5-8.5 for South Africa and Lesotho. We predicted high ecological suitability with low to medium certainty (suitability above 1–3 threshold values) for SINV presence in the Nile Delta, western coast, east, and southern Africa. Under SSP2-4.5 and SSP5-8.5, the presence shrunk in those areas but slightly increased in coastal Nigeria, DRC, and Angola (Fig 6). *Cx. univittattus* (73%) contributed most to SINV suitability, followed by *Cx. pipiens* (9.6%), with minor influence from livestock (1.2%) and human population (0.6%).

We predicted highly suitable habitats for MIDV primarily in South Africa and Lesotho. Under both future scenarios, suitability was stable, albeit with slight declines, notably in South Africa. Current ecological suitability for MIDV presence was highly certain in South Africa, Lesotho, Swaziland, and parts of Eastern and Central Africa. Under future scenarios, high certainty expanded slightly to areas in Egypt, Libya, and Algeria (Fig 6). Key predictors were the mean temperature of the driest quarter (31%) and built-up areas (14%), while *Ma. africana* suitability (1.9%), cropland (1.03%), and human population (0.08%) had minimal impact. Notably, the best model selected for final modeling did not consider *Ae. mcintoshi* among the most important variables.

### Predicting hotspots for risk of zoonotic disease exposure

**Fig 7:**
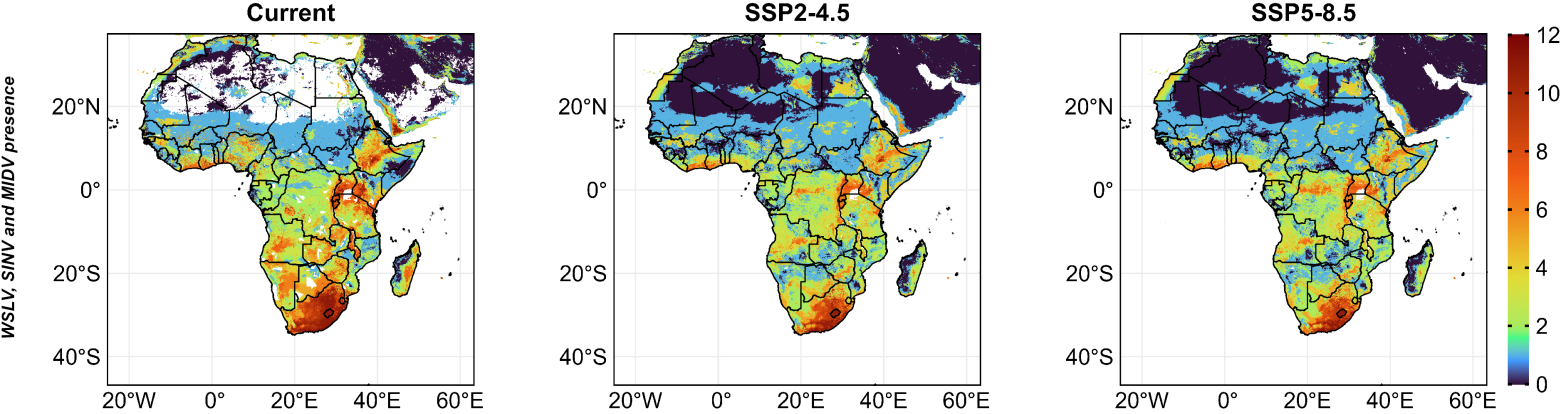
Hotspots for virus exposure risk currently, and during future (2021 – 2040) SSP2-4.5 and SSP5-8.5 scenarios. Risk is based on the highest (12) and the least (0) certainty of virus presence. Africa base layer derived from the openAFRICA “Africa Shapefiles” dataset: https://open.africa/dataset/africa-shapefiles/resource/dcdadd25-0137-4c93-ae5a-82b39d424d60, released under a Creative Commons 4.0 International License.

We analysed WSLV, SINV, and MIDV presence-absence models to map potential exposure risk. High-certainty hotspots (presence scores 9–0) were concentrated in South Africa, Lesotho, Uganda, Kenya, and Ethiopia, while average risk (presence scores 5–8) occurred along the North African coast and parts of SSA. Under SSP2-4.5 and SSP5-8.5, average risk expanded slightly, with low risk potentially emerging in the Sahara (Fig 7).

**Fig 8:**
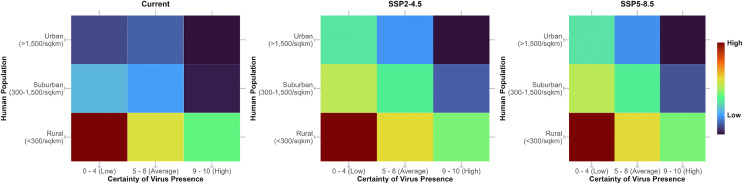
Current, and future (2021 – 2040, SSP2-4.5 and SSP5-8.5) human population density and ecological suitability for potential arboviral exposure. Human population density is presented as people per square kilometre. Ecological suitability represents the combined predicted presence of WSLV, SINV and MIDV. Warmer colors indicate greater overlap between ecological suitability for virus presence and human populations.

We overlaid the combined presence-absence model outputs of the three viruses with gridded human population density across Africa for each scenario. Under current conditions, environmentally suitable areas for low certainty of virus presence (certainty scores 0–4) were more frequently associated with sparsely populated rural areas (<300 people per km^2^), shown in darker red tones, compared with suburban and urban areas, which are predominantly shown in blue (Fig 8). Under future climate scenarios (SSP2-4.5 and SSP5-8.5), environmentally suitable areas for low to moderate certainty of virus presence were projected to expand into increasingly populated suburban and urban settings, illustrated by yellow and green tones (Fig 8).

## Discussion

The recent devastating outbreaks of Zika and Dengue underscore the need to understand mosquito-borne virus distribution under climate change [40,46,89]. This study predicts areas in Africa where current and future ecology may support five vectors (*Ae. circumluteolus, Ae. mcintoshi, Cx. pipiens, Cx. univittatus, and Ma. africana*) and the potential exposure of WSLV, SINV, and MIDV to humans. We present predicted species’ habitat suitability and presence, summarizing the consistency of habitat suitability exceeding multiple threshold criteria.

Our models predict that most SSA countries show variable suitability for the analyzed mosquito species, a finding consistent with mosquito collections since the 1970s which identified arbovirus vectors in multiple countries [42,51]. High suitability is predicted in southern Africa, particularly Lesotho and Northern South Africa, where recent surveys report abundant *Culex*, *Aedes*, and *Mansonia* in conservation areas [42,55]. Unlike dry semi-deserts, conservation areas have more ecological integrity, standing water, and wildlife to support mosquito populations [42]. In arid regions of Kenya, wetlands and seasonal flooding enable breeding for *Ae. mcintoshi* and *Ma. africana* [90,91]. *Ma. africana, Ae. mcintoshi* and *Ae. circumluteolus* are found in savannas and grasslands, while *Cx. pipiens* occurs near lake shores, but also in urban areas, villages and human settlements [51,55,91]. The adaptability of arbovirus vectors across ecosystems highlights the need for diverse entomological surveillance.

*Aedes* species were more tolerant of the coldest and driest seasons compared to *Ma. africana* and *Culex* species, but all mosquitoes preferred maximum temperatures below 32–45°C. In South Africa, *Aedes* species are reported to thrive better between 18–27°C. High temperatures and rainfall typically increase mosquito breeding, serving as an early warning for risk of infection exposure [55,90]. Seasonal mosquito control is essential even during short rains of dry seasons [91]. In addition to climate, built-up areas, human population, and livestock density were key predictors of mosquito habitats. *Ma. Africana, Ae. mcintoshi*, and *Ae. circumluteolus* prefer savanna and grasslands but also thrive in urban areas [42,51,55]. Mosquitoes are adapting to breeding in containers and feeding on livestock and humans near buildings [39]. Urbanization also increases transference risks via mobility and trade [4].

Our virus models did not include niche models for all known mosquito-virus detections; instead, focal vector species were chosen. We acknowledge that omitting other species could bias spatial predictions where those other suspected vectors dominate local transmission cycles. However, detecting a virus in a mosquito does not necessarily establish it as a competent vector in viral maintenance. Notably, for SINV, while enzootic transmission in Europe is sustained mainly by *Cx. torrentium,* in Africa, *Cx. univittatus* is the principal vector with *Cx. pipiens*, *Cx. neavei*, *Cx. quinquefasciatus* and *Cx. perexiguus* playing a supportive role [22,41,54,92,93]. Although WSLV has a broad vector range, enzootic and epizootic cycles rely on floodwater *Aedes* mosquitoes (notably *Ae. circumluteolus* and *Ae. mcintoshi*), which are also the primary vectors in livestock outbreaks after heavy rains [3,9,13,51]. Surveillance studies associate MIDV with several mosquito genera, suggestive of a heterogeneous assemblage of mosquito vectors rather than a single dominant species. However, much of this evidence is based on field isolations rather than controlled vector competence studies [4,32,33,55]. Future studies should explore the relevance of specific floodwater *Aedes* species and *Mansonia* species in MIDV flood-driven transmission, mammal host amplifying and enzootic circulation roles [4,9,28,33]. We also recommend robust mosquito surveys across Africa to investigate the role of different species, local abundance, infection rates and competence, as has been done for SINV in South Africa and Europe [22,54,92,93].

We predicted low ecological suitability for WSLV, SNV, and MIDV presence in Africa, with hotspots in Southern, Eastern, and Western coastal regions. Future scenarios predicted small expansions in North Africa, the Horn of Africa, and the west coast. These findings align with reported sporadic cases and highlight ecological risks for potential human exposure to aid prevention and early warning [3,5,15,30].

We applied the individual niches of the mosquito vectors and included the ecology of known livestock hosts as predictors in our models. The best predictors of the viruses were the ecological niches of their mosquito vectors, a finding corroborated by models of Dengue using its vector, *Ae. aegypti* [57]. However, arboviral disease epidemiology is often complicated by the multitude of vectors, hosts, ecological and human susceptibility factors at work which can’t be fully modelled. Furthermore, unlike Dengue with established primary vectors - *Aedes albopictus* and/or *Aedes aegypti* and humans as the main hosts, the arboviruses in our study are a part of multi-vector multi-host transmission networks that are still under investigation [94]. For example, in Sweden, the seasonal abundance of and SINV prevalence in the main enzootic vector *Cx. torrentium* and in the bridge vector *Ae. cinereus*, are important predictors of outbreaks [24]. Similarly, in South Africa, SINV detections in the enzootic vector *Cx. univittatus* has been linked to increased human infections. However, *Cx. univittatus* and other vectors’ presence alone doesn’t necessarily indicate SINV infection, but incidence is rather seasonal, coinciding with the warm, wet season [21,54]. Furthermore, our study points to the role of climatic predictors in vector and virus ecology, corroborating findings in South Africa linking SINV and Rift Valley Fever (RVF) surges to above-average rainfall and vector breeding [21]. In settings where the evidence for vector-host-virus ecology is underdeveloped, our ecological risk maps can indicate areas that could be targeted for mosquito sampling and virus screening as part of early-warning.

The vectors we analyzed are also important vectors for other viruses of public health importance, i.e., *Ae. circumluteolus* (Spondweni, Zika, Lebombo, Kedougou, Bunyamwera, Ndumu, Pongola and RVF); *Ae*. *mcintoshi* (Babanki, Ndumu, Bunyamwera, Ngari, Pongola, RVF); *Cx. univittatus* (West Nile, Usutu, WSLV, RVF); *Cx. pipiens* (West Nile, Usutu, Semliki Forest, RVF, Japanese Encephalitis, Venezuelan Equine Encephalitis), *Ma. africana* (Spondweni, Usutu, RVF) [4,95,96]. The diversity of niches suitable for these vectors implies that where suitable blood meals or potential hosts (bird, livestock, or human) are present, the transmission, maintenance, and spread of multiple arboviral diseases is likely [4]. Since vector-virus interactions for emerging diseases are often not well understood in the African context, broad mosquito control measures are recommended over species-specific approaches [51,97]. However, mosquito surveillance to detect virus circulation has the best potential for assessing potential risk for human exposure to strengthen early warning and justify resource allocation for intensive control interventions [24,98].

Incorporation of periodic surveys and arbovirus screening into public health systems could improve countries’ knowledge of circulating neglected arboviruses and improve early detection [7,99,100]. In addition, countries should operationalize a One Health approach with multi-sectoral coordinated interventions covering human health, vector control, human activities, animal (wild and domestic) health, as well as ecological monitoring [101]. Countries can leverage existing national and regional policy frameworks such as the Integrated Disease Surveillance and Response (IDSR), and One Health initiatives by the Africa Centers for Disease Control to expand surveillance beyond detection to pre-epidemic multi-host and multi-sectoral surveillance cognizant of ecological risks of arboviral emergence and human exposure risk [102,103].

Our projections suggest a future shift in the spatial overlap between ecological suitability for virus presence and human populations, from predominantly rural settings toward more densely populated urban areas under future socioeconomic and climate scenarios. While this does not imply increased transmission risk per se, it positions human population as another important attribute of ecological transitions that could modify virus presence and human exposure. In Sub-Saharan Africa, rapid population growth and land-use change—characterised by the conversion of forested or wetlands into agricultural, periurban, and built-up environments—may similarly reshape vector habitats and host availability. Such transitions have the potential to modify primarily sylvatic transmission cycles by influencing mosquito behavior, host-seeking patterns, and contact rates between anthropophilic vectors and humans, thereby altering the ecological landscape in which arboviruses persist. [4,35].

Livestock density was an important predictor of vector presence, signalling them as potential amplifying blood meals. In East Africa, *Ae. mcintoshi* and *Ma. africana* abundance were detected in livestock-dense communities where they influenced arboviral activity [41,90]. Syndromic and sentinel surveillance, especially in rural and peri-urban hotspots near livestock, could enhance early detection and understanding of arbovirus transmission and seasonality of risk. Animals like the African Thrushes (for SINV), horses (for SINV and MIDV), and sheep and goats (for WSLV and MIDV) implicated in arboviral maintenance can also contribute to sentinel surveillance [26,27]. For example, SINV sentinel studies in South Africa showed higher pigeon infection risks during warm, wet seasons [54]. Another South African study demonstrated that infected wild and non-equine animals present with neurologic and even fatal SINV infection that could constitute early warnings of cross-species spill overs [27].

Although we evaluated model performance using pROC, AICc, and bootstrap procedures, we acknowledge that bootstrapping with relatively small and spatially clustered species occurrence datasets could have underestimated model uncertainty. Additionally, to address model overfitting due to the limited viral occurrence records, several safeguards were implemented to enhance model robustness. Sampling bias was reduced using a target-group background approach derived from extensive *Aedes*, *Culex*, and *Mansonia* genus occurrence data, a recommended strategy for presence-only species distribution modeling [59,104]. Model complexity was further constrained through conservative regularization, restricted feature classes, variable selection, and AICc-based model evaluation, which explicitly penalizes over-parameterization in small-sample contexts [83]. Predictive performance was assessed using independent testing data, partial ROC, and omission rate thresholds, providing complementary measures of model reliability. Nevertheless, uncertainty remains in regions with sparse data, and model outputs should therefore be interpreted as indicators of ecological suitability for virus presence across ecological scenarios rather than precise outbreak-prone areas.

Many viral pathogens in this study are not notifiable or routinely monitored, limiting case data from many African countries. While serological tests offer basic diagnostic capacity, advanced methods like PCR, virus isolation, and sequencing are more reliable but underutilized due to limited resources, as reflected in the sparse virus sequences in public databases. Despite these challenges, we are confident that we compiled a representative dataset from surveillance, outbreaks, and research, incorporating diverse diagnostic methods and reputable sources. Additionally, we did not have access to a suitable dataset to represent the hosts that influence arboviral transmission, and we only included livestock as a predictor in our models. Where data are available, comprehensive models that account for stochastic introduction events, host susceptibility, host biting, and transmission rates could improve risk mapping [105,106].

Arboviral transmission depends on complex and dynamic processes—including vector–host interactions, vector competence, pathogen prevalence, immunity, and human mobility—that cannot be fully captured using presence-only ecological models. In this study, ecological suitability maps are intended to represent ecological conditions associated with documented virus occurrence rather than realized transmission or infection risk. Similar approaches have been used to map potential ecological suitability or exposure to viruses with complex or poorly resolved transmission systems, including Zika, Ebola Virus, and Leishmaniases [47,107,108]. Given the incomplete knowledge of vector competence and host amplification for many arboviruses, these outputs should be interpreted as screening tools to guide surveillance and ecological inquiry, not as definitive predictors of disease transmission.

## Conclusion

This study presents a continental-scale assessment of the ecological suitability of selected African arboviruses and their mosquito vectors under current and future climatic and environmental conditions. Using presence-only data and macroecological modeling, we identify areas where ecological conditions may support virus presence and where potential human exposure could occur, while clearly distinguishing these patterns from realized transmission risk. The results reveal pronounced spatial heterogeneity and projected shifts in suitability, highlighting the influence of ecological change on arbovirus landscapes.

These outputs should be interpreted as screening tools rather than predictors of disease occurrence. Given the complex, multi-host and multi-vector transmission systems of most zoonotic arboviruses, the maps provide a baseline to guide surveillance, entomological studies, and ecological investigations in data-limited regions. Future integration of vector competence, host dynamics, and epidemiological data will be essential to translate ecological suitability into refined assessments of transmission risk.

## Supporting information

S1 Table Mosquito host per virus species identified in literature. (DOCX)

S2 Table Sources of presence points of the mosquito and virus species. (DOCX)

S3 Table Source datasets for the ecological variables. (DOCX)

S4 Table Model settings and results per species. (DOCX)

S5 Table Area of mosquito species habitat suitability and percent change per scenario. (DOCX)

S1 Fig Correlation matrix of ecological variables. (PNG)

## References

[1] Jones KE, Patel NG, Levy MA, Storeygard A, Balk D, Gittleman JL, et al. Global trends in emerging infectious diseases. Nature. 2008;451(7181):990–3. doi: 10.1038/nature06536 18288193 PMC5960580

[2] Weiss KEH, Alexander RA, Clark R, Louw JG, De Kock VE. Wesselsbron virus - a virus not previously described, associated with abortion in domestic animals. Onderstepoort Journal of Veterinary Research. 1956;27.

[3] Weyer J, Thomas J, Leman PA, Grobbelaar AA, Kemp A, Paweska JT. Human cases of Wesselsbron disease, South Africa 2010-2011. Vector Borne Zoonotic Dis. 2013;13(5):330–6. doi: 10.1089/vbz.2012.1181 23473219

[4] Braack L, Gouveia de Almeida AP, Cornel AJ, Swanepoel R, de Jager C. Mosquito-borne arboviruses of African origin: review of key viruses and vectors. Parasit Vectors. 2018;11(1):29. doi: 10.1186/s13071-017-2559-9 29316963 PMC5759361

[5] Diagne MM, Faye M, Faye O, Sow A, Balique F, Sembène M, et al. Emergence of Wesselsbron virus among black rat and humans in Eastern Senegal in 2013. One Health. 2017;3:23–8. doi: 10.1016/j.onehlt.2017.02.001 28616499 PMC5454166

[6] Henderson BE, Kirya GB, Hewitt LE. Serological survey for arboviruses in Uganda, 1967-69. Bulletin of the World Health Organization. 1970;42(5):797–805. 5311064 PMC2427494

[7] Kokernot RH, Szlamp EL, Levitt J, McIntosh BM. Survey for antibodies against arthropod-borne viruses in the sera of indigenous residents of the Caprivi Strip and Bechuanaland Protectorate. Trans R Soc Trop Med Hyg. 1965;59(5):553–62. doi: 10.1016/0035-9203(65)90158-6 5893148

[8] Diallo M, Nabeth P, Ba K, Sall AA, Ba Y, Mondo M, et al. Mosquito vectors of the 1998-1999 outbreak of Rift Valley Fever and other arboviruses (Bagaza, Sanar, Wesselsbron and West Nile) in Mauritania and Senegal. Med Vet Entomol. 2005;19(2):119–26. doi: 10.1111/j.0269-283X.2005.00564.x 15958020

[9] Jupp PG, Kemp A. Studies on an outbreak of Wesselsbron virus in the Free State Province, South Africa. J Am Mosq Control Assoc. 1998;14(1):40–5. 9599322

[10] Villinger J, Mbaya MK, Ouso D, Kipanga PN, Lutomiah J, Masiga DK. Arbovirus and insect-specific virus discovery in Kenya by novel six genera multiplex high-resolution melting analysis. Mol Ecol Resour. 2017;17(3):466–80. doi: 10.1111/1755-0998.12584 27482633

[11] Heymann CS, Kokernot RH, De Meillon B. Wesselsbron virus infections in man. S Afr Med J. 1958;32(21):543–5. 13556130

[12] Kayiwa JT, Mayanja MN, Nakayiki TM, Senfuka F, Mugga J, Koehler JW, et al. Phylogenetic Analysis of Wesselsbron Virus Isolated from Field-Captured Mosquitoes during a Rift Valley Fever Outbreak in Kabale District, Uganda-2016. Am J Trop Med Hyg. 2022;108(1):161–4. doi: 10.4269/ajtmh.22-0481 36410326 PMC9833084

[13] Eibner GJ, Graff SL, Hieke C, Ochieng JR, Kopp A, Drosten C, et al. Genotypic and phylogeographic insights into a pre-epidemic variant of Wesselsbron virus detected in sylvatic Aedes mcintoshi from Semuliki Forest, Uganda. Microbiol Spectr. 2024;12(12):e0091424. doi: 10.1128/spectrum.00914-24 39530699 PMC11619370

[14] Swanepoel R, Burt FJ. Flaviviruses of Veterinary Importance. In: Mahy BWJ, Van Regenmortel MHV. Encyclopedia of Virology (Third Edition). Oxford: Academic Press. 2008. 234–41.

[15] European Centre for Disease Prevention and Control E. Facts about Sindbis fever. 2023. https://www.ecdc.europa.eu/en/sindbis-fever/facts

[16] Taylor RM, Hurlbut HS, Work TH, Kingston JR, Frothingham TE. Sindbis virus: a newly recognized arthropodtransmitted virus. Am J Trop Med Hyg. 1955;4(5):844–62. doi: 10.4269/ajtmh.1955.4.844 13259009

[17] Storm N, Weyer J, Markotter W, Leman PA, Kemp A, Nel LH, et al. Phylogeny of Sindbis virus isolates from South Africa. Southern African Journal of Epidemiology and Infection. 2013;28(4):207–14. doi: 10.1080/10158782.2013.11441552

[18] Adouchief S, Smura T, Sane J, Vapalahti O, Kurkela S. Sindbis virus as a human pathogen-epidemiology, clinical picture and pathogenesis. Rev Med Virol. 2016;26(4):221–41. doi: 10.1002/rmv.1876 26990827

[19] Brummer-Korvenkontio M, Vapalahti O, Kuusisto P, Saikku P, Manni T, Koskela P, et al. Epidemiology of Sindbis virus infections in Finland 1981-96: possible factors explaining a peculiar disease pattern. Epidemiol Infect. 2002;129(2):335–45. doi: 10.1017/s0950268802007409 12403109 PMC2869892

[20] Lundström JO, Hesson JC. Ockelbo Disease in Sweden: Unraveling the Epidemiology, Ecology, and Evolution of Sindbis Virus. History of Arbovirology: Memories from the Field. Springer Nature Switzerland. 2023. 289–312. doi: 10.1007/978-3-031-22003-6_14

[21] Storm N, Weyer J, Markotter W, Kemp A, Leman PA, Dermaux-Msimang V, et al. Human cases of Sindbis fever in South Africa, 2006-2010. Epidemiol Infect. 2014;142(2):234–8. doi: 10.1017/S0950268813000964 23611492 PMC9151170

[22] Jupp PG, Blackburn NK, Thompson DL, Meenehan GM. Sindbis and West Nile virus infections in the Witwatersrand-Pretoria region. S Afr Med J. 1986;70(4):218–20. 3016922

[23] McIntosh BM, McGillivray GM, Dickinson DB, Malherbe H. Illness caused by sindbis and west nile viruses in South Africa. S Afr Med J. 1964;38:291–4. 14146460

[24] Lundström JO, Hesson JC, Schäfer ML, Östman Ö, Semmler T, Bekaert M, et al. Sindbis virus polyarthritis outbreak signalled by virus prevalence in the mosquito vectors. PLoS Negl Trop Dis. 2019;13(8):e0007702. doi: 10.1371/journal.pntd.0007702 31465453 PMC6738656

[25] Ayhan N, Hachid A, Thirion L, Benallal KE, Pezzi L, Khardine FA, et al. Detection and Isolation of Sindbis Virus from Field Collected Mosquitoes in Timimoun, Algeria. Viruses. 2022;14(5):894. doi: 10.3390/v14050894 35632636 PMC9144192

[26] Matthew DA, Karlsson E, Izang JA, Isberg L, Näslund J, Sjödin A, et al. First detection of Sindbis virus in wild birds in Nigeria. Sci Rep. 2025;15(1):24621. doi: 10.1038/s41598-025-10556-3 40634439 PMC12241308

[27] Steyn J, Fourie I, Steyl J, Williams J, Stivaktas V, Botha E, et al. Zoonotic Alphaviruses in Fatal and Neurologic Infections in Wildlife and Nonequine Domestic Animals, South Africa. Emerg Infect Dis. 2020;26(6):1182–91. doi: 10.3201/eid2606.191179 32441633 PMC7258481

[28] Jupp AJ, Thompson DL, Cornel. Isolations of Middelburg virus from Aedes (Ochlerotatus) juppi McIntosh (Diptera: Culicidae) suggestive of a reservoir vector. A J Afr Entomol. 1987;50(2):393–7. doi: 10.10520/AJA00128789_4310

[29] Kokernot RH, De Meillon B, Paterson HE, Heymann CS, Smithburn KC. Middelburg virus; a hitherto unknown agent isolated from Aedes mosquitoes during an epizootic in sheep in the eastern Cape Province. S Afr J Med Sci. 1957;22(4):145–53. 13529199

[30] Prevention CFDC. Middelburg USA: Centers for Disease Control and Prevention. 2024. https://wwwn.cdc.gov/Arbocat/VirusDetails.aspx?ID=298

[31] Fourie I, Williams J, Ismail A, Jansen van Vuren P, Stoltz A, Venter M. Detection and genome characterization of Middelburg virus strains isolated from CSF and whole blood samples of humans with neurological manifestations in South Africa. PLoS Negl Trop Dis. 2022;16(1):e0010020. doi: 10.1371/journal.pntd.0010020 34979534 PMC8722727

[32] Guarido MM, Fourie I, Meno K, Mendes A, Riddin MA, MacIntyre C, et al. Alphaviruses Detected in Mosquitoes in the North-Eastern Regions of South Africa, 2014 to 2018. Viruses. 2023;15(2):414. doi: 10.3390/v15020414 36851627 PMC9965626

[33] Graff SL, Eibner GJ, Ochieng JR, Jones TC, Nsubuga AM, Lutwama JJ, et al. Detection of two alphaviruses: Middelburg virus and Sindbis virus from enzootic amplification cycles in southwestern Uganda. Front Microbiol. 2024;15:1394661. doi: 10.3389/fmicb.2024.1394661 38863760 PMC11165182

[34] Attoui H, Sailleau C, Mohd Jaafar F, Belhouchet M, Biagini P, Cantaloube JF, et al. Complete nucleotide sequence of Middelburg virus, isolated from the spleen of a horse with severe clinical disease in Zimbabwe. J Gen Virol. 2007;88(Pt 11):3078–88. doi: 10.1099/vir.0.83076-0 17947533

[35] Weaver SC, Barrett ADT. Transmission cycles, host range, evolution and emergence of arboviral disease. Nat Rev Microbiol. 2004;2(10):789–801. doi: 10.1038/nrmicro1006 15378043 PMC7097645

[36] Serena M, Claudia Maria T, Emanuele M. Emerging and re-emerging arboviral diseases as a global health problem. In: Md. Anwarul Azim M, Russell K, Sayeeda R. Public Health. Rijeka: IntechOpen. 2018.

[37] Kopp A, Gillespie TR, Hobelsberger D, Estrada A, Harper JM, Miller RA, et al. Provenance and geographic spread of St. Louis encephalitis virus. mBio. 2013;4(3):e00322-13. doi: 10.1128/mBio.00322-13 23760463 PMC3685209

[38] European Centre for Disease Prevention and Control E Aedes aegypti - Factsheet for experts. 2023. https://www.ecdc.europa.eu/en/disease-vectors/facts/mosquito-factsheets/aedes-aegypti#:~:text=Current%20issues%201%20Invasive%20species%20The%20invasive%20success,dengue%20virus%20chikungunya%20virus%20and%20Zika%20virus.%20

[39] Powell JR. Mosquito-Borne Human Viral Diseases: Why Aedes aegypti?. Am J Trop Med Hyg. 2018;98(6):1563–5. doi: 10.4269/ajtmh.17-0866 29557341 PMC6086192

[40] Baker RE, Mahmud AS, Miller IF, Rajeev M, Rasambainarivo F, Rice BL, et al. Infectious disease in an era of global change. Nat Rev Microbiol. 2022;20(4):193–205. doi: 10.1038/s41579-021-00639-z 34646006 PMC8513385

[41] Ochieng C, Lutomiah J, Makio A, Koka H, Chepkorir E, Yalwala S, et al. Mosquito-borne arbovirus surveillance at selected sites in diverse ecological zones of Kenya; 2007 - 2012. Virol J. 2013;10:140. doi: 10.1186/1743-422X-10-140 23663381 PMC3669043

[42] Cornel AJ, Lee Y, Almeida APG, Johnson T, Mouatcho J, Venter M, et al. Mosquito community composition in South Africa and some neighboring countries. Parasit Vectors. 2018;11(1):331. doi: 10.1186/s13071-018-2824-6 29859109 PMC5984792

[43] Mossel EC, Crabtree MB, Mutebi J-P, Lutwama JJ, Borland EM, Powers AM, et al. Arboviruses Isolated From Mosquitoes Collected in Uganda, 2008-2012. J Med Entomol. 2017;54(5):1403–9. doi: 10.1093/jme/tjx120 28874015 PMC5968633

[44] Sigei F, Nindo F, Mukunzi S, Ng’ang’a Z, Sang R. Evolutionary analyses of Sindbis virus strains isolated from mosquitoes in Kenya. Arch Virol. 2018;163(9):2465–9. doi: 10.1007/s00705-018-3869-8 29781064

[45] Jentes ES, Poumerol G, Gershman MD, Hill DR, Lemarchand J, Lewis RF, et al. The revised global yellow fever risk map and recommendations for vaccination, 2010: consensus of the Informal WHO Working Group on Geographic Risk for Yellow Fever. Lancet Infect Dis. 2011;11(8):622–32. doi: 10.1016/S1473-3099(11)70147-5 21798462

[46] Bhatt S, Gething PW, Brady OJ, Messina JP, Farlow AW, Moyes CL, et al. The global distribution and burden of dengue. Nature. 2013;496(7446):504–7. doi: 10.1038/nature12060 23563266 PMC3651993

[47] Messina JP, Kraemer MU, Brady OJ, Pigott DM, Shearer FM, Weiss DJ, et al. Mapping global environmental suitability for Zika virus. Elife. 2016;5:e15272. doi: 10.7554/eLife.15272 27090089 PMC4889326

[48] Shapiro LLM, Whitehead SA, Thomas MB. Quantifying the effects of temperature on mosquito and parasite traits that determine the transmission potential of human malaria. PLoS Biol. 2017;15(10):e2003489. doi: 10.1371/journal.pbio.2003489 29036170 PMC5658182

[49] National Center for Biotechnology Information. NCBI Virus. 2024. https://www.ncbi.nlm.nih.gov/labs/virus/vssi/#/virus?SeqType_s=Nucleotide

[50] Google. Google Maps. 2024. https://www.google.com/maps

[51] McIntosh BM, Jupp PG, De Sousa J. Further isolations of the arboviruses from mosquitoes collected in Tongaland, South Africa, 1960-1968. J Med Entomol. 1972;9(2):155–9. doi: 10.1093/jmedent/9.2.155 4402531

[52] M’ghirbi Y, Mousson L, Moutailler S, Lecollinet S, Amaral R, Beck C, et al. West Nile, Sindbis and Usutu Viruses: Evidence of Circulation in Mosquitoes and Horses in Tunisia. Pathogens. 2023;12(3):360. doi: 10.3390/pathogens12030360 36986282 PMC10056592

[53] Gutiérrez-López R, Ruiz-López MJ, Ledesma J, Magallanes S, Nieto C, Ruiz S, et al. First isolation of the Sindbis virus in mosquitoes from southwestern Spain reveals a new recent introduction from Africa. One Health. 2024;20:100947. doi: 10.1016/j.onehlt.2024.100947 39760017 PMC11699435

[54] McIntosh BM, Jupp PG. Infections in sentinel pigeons by Sindbis and West Nile viruses in South Africa, with observations on Culex (Culex) univittatus (Diptera: Culicidae) attracted to these birds. J Med Entomol. 1979;16(3):234–9. doi: 10.1093/jmedent/16.3.234 537007

[55] Guarido MM, Riddin MA, Johnson T, Braack LEO, Schrama M, Gorsich EE, et al. Aedes species (Diptera: Culicidae) ecological and host feeding patterns in the north-eastern parts of South Africa, 2014-2018. Parasit Vectors. 2021;14(1):339. doi: 10.1186/s13071-021-04845-9 34174956 PMC8235819

[56] Global Biodiversity Information Facility GOU. Aedes circumluteolus (Theobald, 1908) occurrence download. The Global Biodiversity Information Facility. 2024.

[57] Johnson EE, Escobar LE, Zambrana-Torrelio C. An Ecological Framework for Modeling the Geography of Disease Transmission. Trends Ecol Evol. 2019;34(7):655–68. doi: 10.1016/j.tree.2019.03.004 31078330 PMC7114676

[58] Sofia M, Giannakopoulos A, Giantsis IA, Touloudi A, Birtsas P, Papageorgiou K, et al. West Nile Virus Occurrence and Ecological Niche Modeling in Wild Bird Species and Mosquito Vectors: An Active Surveillance Program in the Peloponnese Region of Greece. Microorganisms. 2022;10(7):1328. doi: 10.3390/microorganisms10071328 35889046 PMC9320058

[59] Phillips SJ, Anderson RP, Schapire RE. Maximum entropy modeling of species geographic distributions. Ecological Modelling. 2006;190(3–4):231–59. doi: 10.1016/j.ecolmodel.2005.03.026

[60] Fick SE, Hijmans RJ. WorldClim 2: new 1‐km spatial resolution climate surfaces for global land areas. Intl Journal of Climatology. 2017;37(12):4302–15. doi: 10.1002/joc.5086

[61] toIPCC. Summary for Policymakers. Synthesis Report. Contribution of Working Groups I, II and III to the sixth assessment report of the Intergovernmental Panel on Climate Change. Geneva, Switzerland: IPCC. 2023.

[62] Ayugi B, Ngoma H, Babaousmail H, Karim R, Iyakaremye V, Lim Kam Sian KTC, et al. Evaluation and projection of mean surface temperature using CMIP6 models over East Africa. Journal of African Earth Sciences. 2021;181:104226. doi: 10.1016/j.jafrearsci.2021.104226

[63] Programme WCR. 2021. https://esgf-node.ipsl.upmc.fr/projects/cmip6-ipsl/

[64] Gridded Livestock Density (Global - 2015 - 10 km). NSAL FaAO-. 2022.

[65] NSAL FAO. Gridded livestock density (Global - 2020 - 10 km). 2024.

[66] Joint Research Centre - JRC - European Commission, Center for International Earth Science Information Network - CIESIN - Columbia University. Global human settlement layer: Population and built-up estimates, and degree of urbanization settlement model grid. Palisades, New York: NASA Socioeconomic Data and Applications Center (SEDAC). 2021.

[67] Florczyk AJ, Ehrlich D, Freire S, Kemper T, Maffenini L, Melchiorri M. Luxembourg: Publications Office of the European Union. 2019. doi: 20240916

[68] Wang X, Meng X, Long Y. Projecting 1 km-grid population distributions from 2020 to 2100 globally under shared socioeconomic pathways. figshare. 2022. 10.1038/s41597-022-01675-x PMC9466344 36097271

[69] Potapov P, Turubanova S, Hansen MC, Tyukavina A, Zalles V, Khan A, et al. Global maps of cropland extent and change show accelerated cropland expansion in the twenty-first century. Nat Food. 2022;3(1):19–28. doi: 10.1038/s43016-021-00429-z 37118483

[70] Chen M, Vernon CR, Graham NT, Hejazi M, Huang M, Cheng Y, et al. Global land use for 2015-2100 at 0.05° resolution under diverse socioeconomic and climate scenarios. Sci Data. 2020;7(1):320. doi: 10.1038/s41597-020-00669-x 33009403 PMC7532189

[71] Friedl M, Sulla-Menashe D. MODIS/Terra Aqua Land Cover Type Yearly L3 Global 500m SIN Grid V061. NASA EOSDIS Land Processes Distributed Active Archive Center. 2022.

[72] Team A. Application for extracting and exploring analysis ready samples (AppEEARS). 3.52 ed. 2024.

[73] Didan K. MODIS/Terra vegetation indices monthly L3 global 1km SIN grid V061. In: Center NELPDAA. 2021.

[74] de Oliveira SV, Escobar LE, Peterson AT, Gurgel-Gonçalves R. Potential geographic distribution of hantavirus reservoirs in Brazil. PLoS One. 2013;8(12):e85137. doi: 10.1371/journal.pone.0085137 24391989 PMC3877355

[75] Elith J, Graham C, Anderson RP, Dudík M, Ferrier S, Guisan A, et al. Novel methods improve prediction of species’ distributions from occurrence data. Ecography. 2006;29(2):129–51. doi: 10.1111/j.2006.0906-7590.04596.x

[76] Hijmans RJ. Terra: Spatial data analysis. 2024.

[77] Pebesma E, Bivand R. Spatial Data Science: With Applications in R. Chapman and Hall/CRC. 2023.

[78] Team RC. R: A Language and Environment for Statistical Computing. 4.4.1 ed. Vienna, Austria: R Foundation for Statistical Computing. 2024.

[79] Guisan A, Thuiller W, Zimmermann NE. Habitat Suitability and Distribution Models: With Applications in R. Cambridge: Cambridge University Press. 2017.

[80] Phillips SJ, Schapire RE. Maxent software for modeling species niches and distributions. 2024.

[81] Elith J, Kearney M, Phillips S. The art of modelling range-shifting species. Methods in Ecology and Evolution. 2010;1(4):330–42. doi: 10.1111/j.2041-210x.2010.00036.x

[82] Barber RA, Ball SG, Morris RKA, Gilbert F. Target‐group backgrounds prove effective at correcting sampling bias in Maxent models. Diversity and Distributions. 2021;28(1):128–41. doi: 10.1111/ddi.13442

[83] Cobos ME, Peterson AT, Barve N, Osorio-Olvera L. kuenm: an R package for detailed development of ecological niche models using Maxent. PeerJ. 2019;7:e6281. doi: 10.7717/peerj.6281 30755826 PMC6368831

[84] Peterson AT, Papeş M, Soberón J. Rethinking receiver operating characteristic analysis applications in ecological niche modeling. Ecological Modelling. 2008;213(1):63–72. doi: 10.1016/j.ecolmodel.2007.11.008

[85] Anderson RP, Lew D, Peterson AT. Evaluating predictive models of species’ distributions: criteria for selecting optimal models. Ecological Modelling. 2003;162(3):211–32. doi: 10.1016/s0304-3800(02)00349-6

[86] Wickham H. ggplot2: Elegant Graphics for Data Analysis. New York: Springer-Verlag. 2016.

[87] Garnier SNR, Rudis R, Camargo AP, Sciaini M, Scherer C. viridis (Lite) - Colorblind-friendly color maps for R. 2024.

[88] Auguie B, Antonov A. gridExtra: miscellaneous functions for “grid” graphics. 2017.

[89] Talero-Gutiérrez C, Rivera-Molina A, Pérez-Pavajeau C, Ossa-Ospina I, Santos-García C, Rojas-Anaya MC, et al. Zika virus epidemiology: from Uganda to world pandemic, an update. Epidemiol Infect. 2018;146(6):673–9. doi: 10.1017/S0950268818000419 29536828 PMC9134366

[90] Arum SO, Weldon CW, Orindi B, Landmann T, Tchouassi DP, Affognon HD, et al. Distribution and diversity of the vectors of Rift Valley fever along the livestock movement routes in the northeastern and coastal regions of Kenya. Parasit Vectors. 2015;8:294. doi: 10.1186/s13071-015-0907-1 26018134 PMC4449603

[91] Lutomiah J, Bast J, Clark J, Richardson J, Yalwala S, Oullo D, et al. Abundance, diversity, and distribution of mosquito vectors in selected ecological regions of Kenya: public health implications. J Vector Ecol. 2013;38(1):134–42. doi: 10.1111/j.1948-7134.2013.12019.x 23701618

[92] Jupp PG, McIntosh BM. Quantitative experiments on the vector capability of Culex (Culex) univittatus Theobald with West Nile and Sindbis viruses. J Med Entomol. 1970;7(3):371–3. doi: 10.1093/jmedent/7.3.371 5451049

[93] Hesson JC, Verner-Carlsson J, Larsson A, Ahmed R, Lundkvist Å, Lundström JO. Culex torrentium Mosquito Role as Major Enzootic Vector Defined by Rate of Sindbis Virus Infection, Sweden, 2009. Emerg Infect Dis. 2015;21(5):875–8. doi: 10.3201/eid2105.141577 25898013 PMC4412225

[94] Diaz LA, Flores FS, Quaglia A, Contigiani MS. Intertwined arbovirus transmission activity: reassessing the transmission cycle paradigm. Front Physiol. 2013;3:493. doi: 10.3389/fphys.2012.00493 23335900 PMC3542535

[95] Unit WRB. Aedes mcintoshi species page. 2021. http://wrbu.si.edu/vectorspecies/mosquitoes/mcintoshi

[96] Unit WRB. Culex pipiens species page. 2021. http://wrbu.si.edu/vectorspecies/mosquitoes/pipiens

[97] Omondi D, Masiga DK, Ajamma YU, Fielding BC, Njoroge L, Villinger J. Unraveling Host-Vector-Arbovirus Interactions by Two-Gene High Resolution Melting Mosquito Bloodmeal Analysis in a Kenyan Wildlife-Livestock Interface. PLoS One. 2015;10(7):e0134375. doi: 10.1371/journal.pone.0134375 26230507 PMC4521840

[98] Kilpatrick AM, Pape WJ. Predicting human West Nile virus infections with mosquito surveillance data. Am J Epidemiol. 2013;178(5):829–35. doi: 10.1093/aje/kwt046 23825164 PMC3755645

[99] Koopmans M. Surveillance strategy for early detection of unusual infectious disease events. Curr Opin Virol. 2013;3(2):185–91. doi: 10.1016/j.coviro.2013.02.003 23612329 PMC7102709

[100] Mandl KD, Overhage JM, Wagner MM, Lober WB, Sebastiani P, Mostashari F, et al. Implementing syndromic surveillance: a practical guide informed by the early experience. J Am Med Inform Assoc. 2004;11(2):141–50. doi: 10.1197/jamia.M1356 14633933 PMC353021

[101] Mensah EA, Gyasi SO, Nsubuga F, Alali WQ. A proposed One Health approach to control yellow fever outbreaks in Uganda. One Health Outlook. 2024;6(1):9. doi: 10.1186/s42522-024-00103-x 38783349 PMC11119388

[102] CDC A. Framework for One Health Practice in National Public Health Institutes: Zoonotic Disease Prevention and Control. African Union. 2020.

[103] Health Mo. National Technical Guidelines for Integrated Disease Surveillance and Response. Kampala, Uganda. 2021.

[104] Tong R, Yesson C, Yu J, Luo Y, Zhang L. Key factors for species distribution modeling in benthic marine environments. Front Mar Sci. 2023;10. doi: 10.3389/fmars.2023.1222382

[105] Guerrero BV, Steindorf V, Blasco-Aguado R, Mateus L, Cevidanes A, Barandika JF, et al. Assessing the spatio-temporal risk of Aedes-borne arboviral diseases in non-endemic regions: The case of Northern Spain. PLoS Negl Trop Dis. 2025;19(7):e0013325. doi: 10.1371/journal.pntd.0013325 40720537 PMC12313078

[106] Brady OJ, Bastos LS, Caldwell JM, Cauchemez S, Clapham HE, Dorigatti I, et al. Why the growth of arboviral diseases necessitates a new generation of global risk maps and future projections. PLoS Comput Biol. 2025;21(4):e1012771. doi: 10.1371/journal.pcbi.1012771 40184562 PMC11970912

[107] Pigott DM, Bhatt S, Golding N, Duda KA, Battle KE, Brady OJ, et al. Global distribution maps of the leishmaniases. Elife. 2014;3:e02851. doi: 10.7554/eLife.02851 24972829 PMC4103681

[108] Pigott DM, Golding N, Mylne A, Huang Z, Henry AJ, Weiss DJ, et al. Mapping the zoonotic niche of Ebola virus disease in Africa. Elife. 2014;3:e04395. doi: 10.7554/eLife.04395 25201877 PMC4166725

