# Supplementary material for: Modeling the potential distribution of Wesselsbron, Sindbis, and Middelburg viruses and their vectors in Africa under future climatic and land-use changes
Source: PLoS Negl Trop Dis. 2026 Mar 4;20(3):e0014072. doi: 10.1371/journal.pntd.0014072 (PMC12970976; doi:10.1371/journal.pntd.0014072)
Supplement: S1 Table — (DOCX) [file pntd.0014072.s001.docx]

**S1 Table:** **Mosquito host species per virus species identified in literature, known vector importance, GBIF search results, and final counts that were available for vector models**

*Vector importance as mentioned in the literature and in the absence of vector competence data

**Post-cleaning counts are the unique presence points and include data from the Uganda mosquito survey

| **Mosquito host named in literature** | ***WSLV*** | ***SINV*** | ***MIDV*** | **Total in dataset** | ***Important vector?** | **GBIF search (with coordinates)** | ****Count post-cleaning/exclusion criteria** |
| --- | --- | --- | --- | --- | --- | --- | --- |
| *Culex univittatus* | 0 | 15 | 1 | 16 | Yes (SINV), Unclear (MIDV) | 942 | 172 |
| *Culicidae* | 12 | 0 | 0 | 12 | Species missing | Not species | N/A |
| *Aedes circumluteolus* | 6 | 1 | 1 | 8 | Yes (WSLV), Suspected (MIDV) | 62 | 121 |
| *Aedes vexans* | 7 | 0 | 0 | 7 | Yes (WSLV) | 52 | Insufficient points |
| *Aedes (Ochlerotatus) caballus =* *Ochlerotatus caballus (Theobald, 1912)  = Aedes caballus (Theobald, 1912)* | 2 | 0 | 1 | 3 | Yes (WSLV), Suspected (MIDV) | 30 | 10  *Ae. mcintoshi* and *Ae. circumluteolus* preferred |
| *Aedes mcintoshi* | 4 | 0 | 1 | 5 | Yes (WSLV), Suspected (MIDV) | 215 | 89 |
| *Aedes durbanensis* | 0 | 1 | 1 | 2 | Suspected (MIDV) | 2 | Insufficient points |
| *Culex pipiens* | 0 | 4 | 0 | 4 | Yes (SINV) | 3,878 | 141 |
| *Aedes meigen* | 2 | 0 | 0 | 2 | Species missing | Not species | N/A |
| *Mansonia uniformis* | 1 | 2 | 0 | 3 | Suspected (WSLV) | 275 | *Ae. mcintoshi* and *Ae. circumluteolus* preferred |
| *Mansonia africana* | 0 | 0 | 1 | 1 | Suspected (MIDV) | 258 | 157 |
| *Culex spp* | 2 | 3 | 0 | 5 | Species missing | Not species | N/A |
| *Aedes dalzieli* | 2 | 0 | 0 | 2 | Suspected (WSLV) | 10 | Insufficient points |
| *Aedes ochraceus (Theobald)* | 1 | 1 | 0 | 2 | Suspected (WSLV) | 87 | *Ae. mcintoshi* and *Ae. circumluteolus* preferred |
| *Anopheles coustani* | 1 | 0 | 1 | 2 | Suspected (WSLV) | 321 | *Ae. mcintoshi* and *Ae. circumluteolus* preferred |
| *Aedes dentatus* | 0 | 0 | 1 | 1 | Suspected (MIDV) | 16 | Insufficient points |
| *Aedes vittatus* | 0 | 0 | 1 | 1 | Unclear (MIV) | 80 | N/A |
| *Culex cinereus* | 0 | 1 | 0 | 1 | Yes (SINV) | 162 | *Cx. pipiens* preferred |
| *Aedes gibbinsi* | 1 | 0 | 0 | 1 | Unclear | 0 | N/A |
| *Aedes africanus = Stegomyia africanus* | 1 | 0 | 0 | 1 | Unclear | 217 | N/A |
| *Aedes tricholabis* | 1 | 2 | 0 | 3 | Yes (WSLV) | 1 | *Ae. mcintoshi* and *Ae. circumluteolus* preferred |
| *Culex terzii* | 0 | 0 | 1 | 1 | Unclear (MIDV) | 8 | N/A |
| *Eretmapodites chrysogaster* | 0 | 0 | 1 | 1 | Suspected (MIDV) | 58 | N/A |
| *Aedes tarsalis* | 0 | 1 | 0 | 1 | Unclear | 11 | N/A |
| *Aedes tricholabis* | 1 | 2 | 0 | 3 | Yes (WSLV) | 1 | *Ae. mcintoshi* and *Ae. circumluteolus* preferred |
| *Anopheles gambiae* | 0 | 1 | 0 | 1 | Unclear | 118195 | N/A |
| *Coquelittidia fuscopennata = Mansonia fuscopennata* | 0 | 3 | 0 | 3 | Suspected (SINV) | 26 | *Cx. pipiens* preferred |
| *Culex annulioris* | 0 | 1 | 0 | 1 | Suspected (SINV) | 75 | *Cx. pipiens* preferred |
| *Culex perfuscus* | 0 | 1 | 0 | 1 | Suspected (SINV) | 11 | *Cx. pipiens* preferred |
| *Culex poicilipes* | 0 | 1 | 0 | 1 | Suspected (SINV) | 76 | *Cx. pipiens* preferred |
| *Culex theileri* | 0 | 1 | 0 | 1 | Suspected (SINV) | 486 | *Cx. pipiens* preferred |
| *Culex tigripes = Lutzia tigripes* | 0 | 1 | 0 | 1 | Suspected (SINV) | 19 | *Cx. pipiens* preferred |
| *Culex vansomereni* | 0 | 1 | 0 | 1 | Suspected (SINV) | 8 | *Cx. pipiens* preferred |
| *Culex zombaensis* | 0 | 1 | 0 | 1 | Suspected (SINV) | 3 | *Cx. pipiens* preferred |
| *Aedes juppi = Ochlerotatus juppi (McIntosh, 1973)* | 1 | 1 | 2 | 4 | Yes (WSLV), Suspected (MIDV) | 3 | Insufficient points |
| *Culex torrentium* | 0 | 0 | 0 | 0 | Yes (SINV) | 1 | Insufficient points |
| *Culex perexiguus* | 0 | 0 | 0 | 0 | Yes (SINV) | 344 | *Cx. pipiens* preferred |
| *Culex quinquefasciatus* | 0 | 0 | 0 | 0 | Yes (SINV) | 325 | *Cx. pipiens* preferred |
| *Culex neavei* | 0 | 1 | 0 | 1 | Yes (SINV) | 30 | *Cx. pipiens* preferred |
| *Aedes cinereus* | 0 | 1 | 0 | 1 | Yes (SINV) | 0 | Bridge vector in Europe |
| *Anopheles maculipennis* | 0 | 0 | 0 | 0 | Yes (SINV) | 38 | *Cx. pipiens* preferred |
| *Culiseta morsitans (Theobald, 1901)* | 0 | 0 | 0 | 0 | Yes (SINV) | 1 | Vector in Europe |
| Totals | 44 | 44 | 13 | 101 |  |  |  |
