# Supplementary material for: Modeling the potential distribution of Wesselsbron, Sindbis, and Middelburg viruses and their vectors in Africa under future climatic and land-use changes
Source: PLoS Negl Trop Dis. 2026 Mar 4;20(3):e0014072. doi: 10.1371/journal.pntd.0014072 (PMC12970976; doi:10.1371/journal.pntd.0014072)
Supplement: S2 Table — (DOCX) [file pntd.0014072.s002.docx]

**S2 Table. Presence points of the mosquito and virus species used in species distribution modeling, time of original sample collection, and references of sources**

| **Species** | **Sample collection time** | **Number of presence points** | **Source references** |
| --- | --- | --- | --- |
| ***Wesselsbron virus***  **(n = 154)** | 2010 – 2011 | 3 | [1] |
|  | 2013 – 2014 | 3 | [2] |
|  | 1955 – 2022 | 17 | [3] |
|  | 2016 | 2 | [4] |
|  | 1955 | 2 | [5] |
|  | 1957 – 2011 | 17 | [6] |
|  | 1956 – 1957 | 6 | [7] |
|  | 1996 | 1 | [8] |
|  | 1964 | 1 | [9] |
|  | 1954 – 1957 | 21 | [10] |
|  | 1984 | 3 | [11] |
|  | 1959 | 12 | [12] |
|  | 1967 – 1969 | 7 | [13] |
|  | 1957 | 1 | [14] |
|  | 1959 | 1 | [15] |
|  | 1957 | 1 | [16] |
|  | 1972 | 1 | [17] |
|  | 1996 | 3 | [8] |
|  | 1992 | 1 | [18] |
|  | 1964 – 1965 | 15 | [19] |
|  | 1999 | 6 | [20] |
|  | 1989 – 1991 | 2 | [21] |
|  | 1972 – 1975 | 4 | [22] |
|  | 1995 | 1 | [23] |
|  | 1977 | 1 | [24] |
|  | 1995 | 1 | [25] |
|  | 2010 | 2 | [26] |
|  | 1996 | 2 | [27] |
|  | 1991 | 3 | [28] |
|  | 1966 – 1968 | 3 | [29] |
|  | 1977 | 2 | [30] |
|  | 1987 | 1 | [31] |
|  | 1975 | 2 | [32] |
|  | 1971 – 1973 | 2 | [33] |
|  | 1989 | 1 | [34] |
|  | 1970 | 2 | [35] |
|  | 1992 – 1993 | 1 | [36] |
| ***Sindbis virus***  **(n = 109)** | 1972 – 1975 | 2 | [22] |
|  | 1967 – 1969 | 21 | [13] |
|  | 1963 | 1 | [37] |
|  | 1954 | 2 | [38] |
|  | 1963 | 1 | [39] |
|  | 1966 – 1968 | 3 | [29] |
|  | 1977 | 1 | [24] |
|  | 1971 – 1972 | 2 | [33] |
|  | 1954 – 1974 | 3 | [40] |
|  | 1967 – 1971 | 2 | [41] |
|  | 1985 | 1 | [42] |
|  | 1956 | 3 | [10] |
|  | 1962 | 1 | [9] |
|  | 2016 | 1 | [43] |
|  | 2014 – 2018 | 19 | [44] |
|  | 2007 – 2012 | 3 | [45] |
|  | 2019 | 1 | [46] |
|  | 2010 | 1 | [47] |
|  | 2019 | 2 | [48] |
|  | 2007 – 2013 | 4 | [49] |
|  | 2012 | 1 | [50] |
|  | 1984 | 5 | [51] |
|  | 1980 | 6 | [52] |
|  | 1959 | 13 | [12] |
|  | 1971 | 1 | [53] |
|  | 2010 | 2 | [26] |
|  | 2000 – 2003 | 6 | [54] |
|  | 1960 | 1 | [55] |
| ***Middelburg virus* (n = 49)** | 1977 – 1984 | 2 | [24] |
|  | 1981 | 3 | [28] |
|  | 1960 – 1968 | 2 | [9] |
|  | 1993 | 1 | [56] |
|  | 2010 | 2 | [26] |
|  | 2019 | 2 | [48] |
|  | 2014 – 2018 | 32 | [44] |
|  | 1985 | 1 | [42] |
|  | 1996 | 2 | [8] |
|  | 1957 | 1 | [57] |
|  | 1957 | 1 | [10] |
| ***Aedes circumluteolus* (n = 121)** | 2018 – 2019 | 106 | [48] |
|  | 1960 – 2018 | 15 | [58] |
| ***Aedes mcintoshi* (n = 89)** | 2012 | 18 | [59] |
|  | 2012 – 2022 | 34 | [60] |
|  | 2018 – 2019 | 37 | [48] |
| ***Culex univittatus***  **(n = 172)** | 2018 – 2019 | 65 | [48] |
|  | 1900 – 2022 | 107 | [61] |
| ***Culex pipiens***  **(n = 241)** | 2018 – 2019 | 53 | [48] |
|  | 1912 – 2024 | 188 | [62] |
| ***Mansonia africana***  **(n = 157)** | 1911 – 2022 | 100 | [63] |
|  | 2018 – 2019 | 57 | [48] |
| ***Aedes*** ***meigen*** | 1899 – 2024 | 19,797 | [64] |
| ***Culex*** ***linnaeus*** | 1900 – 2024 | 17,530 | [65] |
| ***Mansonia blanchard*** | 1911 – 2024 | 1,423 | [66] |

**References**

1. Villinger J, Mbaya MK, Ouso D, Kipanga PN, Lutomiah J, Masiga DK. Arbovirus and insect-specific virus discovery in Kenya by novel six genera multiplex high-resolution melting analysis. Molecular Ecology Resources. 2017;17(3):466-80. doi: <https://doi.org/10.1111/1755-0998.12584>.

2. Diagne MM, Faye M, Faye O, Sow A, Balique F, Sembène M, et al. Emergence of Wesselsbron virus among black rat and humans in Eastern Senegal in 2013. One health (Amsterdam, Netherlands). 2017;3:23-8. Epub 2017/06/16. doi: 10.1016/j.onehlt.2017.02.001. PubMed PMID: 28616499; PubMed Central PMCID: PMCPMC5454166.

3. NCBI Virus: Wesselsbron virus, taxid:164416 [Internet]. 2024. Available from: <https://www.ncbi.nlm.nih.gov/labs/virus/vssi/#/virus?SeqType_s=Nucleotide&Region_s=Africa&VirusLineage_ss=Wesselsbron%20virus,%20taxid:164416>.

4. Kayiwa JT, Mayanja MN, Nakayiki TM, Senfuka F, Mugga J, Koehler JW, et al. Phylogenetic Analysis of Wesselsbron Virus Isolated from Field-Captured Mosquitoes during a Rift Valley Fever Outbreak in Kabale District, Uganda—2016. The American Journal of Tropical Medicine and Hygiene. 2023;108(1):161-4. doi: 10.4269/ajtmh.22-0481.

5. Weiss KEH, D.A.; Alexander, R.A.; Alexander, R.A.; Clark, R.; Louw, J.G.; De Kock, V.E. Wesselsbron virus - a virus not previously described, associated with abortion in domestic animals. Onderstepoort Journal of Veterinary Research. 1956;27.

6. Weyer J, Thomas J, Leman PA, Grobbelaar AA, Kemp A, Paweska JT. Human Cases of Wesselsbron Disease, South Africa 2010–2011. 2013;13(5):330-6. doi: 10.1089/vbz.2012.1181. PubMed PMID: 23473219.

7. Belonje CWA. Field observations on Wesselsbron disease. 1958;29(1):1-14. doi: doi:10.10520/AJA00382809_725.

8. Jupp PG, Kemp A. Studies on an outbreak of Wesselsbron virus in the Free State Province, South Africa. Journal of the American Mosquito Control Association. 1998;14(1):40-5. PubMed PMID: 9599322.

9. Mclntosh BM, Jupp PG, de Sousa J. Further Isolations of Arboviruses from Mosquitoes Collected in Tongaland, South Africa, 1960–1968. Journal of Medical Entomology. 1972;9(2):155-9. doi: 10.1093/jmedent/9.2.155 %J Journal of Medical Entomology.

10. D. Smithburn KC, Kokernot, R.H., Heymann, C.S., Weinbren, M.P. Zentkowsky. Neutralizing antibodies for certain viruses in the sera of human beings in Northern Natal. 1959;33(27):555-61. doi: doi:10.10520/AJA20785135_39788.

11. Rodhain F, Gonzalez JP, Mercier E, Helynck B, Larouze B, Hannoun C. Arbovirus infections and viral haemorrhagic fevers in Uganda: a serological survey in Karamoja district, 1984. Transactions of The Royal Society of Tropical Medicine and Hygiene. 1989;83(6):851-4. doi: 10.1016/0035-9203(89)90352-0 %J Transactions of The Royal Society of Tropical Medicine and Hygiene.

12. Kokernot RH, Szlamp EL, Levitt J, McIntosh BM. Survey for antibodies against arthropod-borne viruses in the sera of indigenous residents of the caprivi strip and bechuanaland protectorate*. Transactions of The Royal Society of Tropical Medicine and Hygiene. 1965;59(5):553-62. doi: 10.1016/0035-9203(65)90158-6 %J Transactions of The Royal Society of Tropical Medicine and Hygiene.

13. Henderson BE, Kirya GB, Hewitt LE. Serological survey for arboviruses in Uganda, 1967-69. Bulletin of the World Health Organization. 1970;42(5):797-805. Epub 1970/01/01. PubMed PMID: 5311064; PubMed Central PMCID: PMCPMC2427494.

14. B. Heymann CS, Kokernot, R.H. de Meillon. Wesselsbron virus infections in man. 1958;32(21):543-5. doi: doi:10.10520/AJA20785135_38815.

15. Weinbren MJ. Some clinical observations on a human case of infection with Wesselsbron virus. East Afr Virus Res Inst Rep. 1959;9:22-3.

16. Swanepoel R. Wesselsbron virus disease. In: Monath TP, editor. The Arboviruses: Epidemiology and Ecology. 1st ed: CRC Press; 1988. p. 32–57.

17. Tomori O, Monath TP, O'Connor EH, Lee VH, Cropp CB. Arbovirus Infections among Laboratory Personnel in Ibadan, Nigeria %J The American Journal of Tropical Medicine and Hygiene. 1981;30(4):855-61. doi: 10.4269/ajtmh.1981.30.855.

18. Traoré-lamizana M, Fontenille D, Diallo M, Bâ Y, Zeller HG, Mondo M, et al. Arbovirus Surveillance from 1990 to 1995 in the Barkedji Area (Ferlo) of Senegal, a Possible Natural Focus of Rift Valley Fever Virus. Journal of Medical Entomology. 2001;38(4):480-92. doi: 10.1603/0022-2585-38.4.480 %J Journal of Medical Entomology.

19. Pinto MR. Survey for antibodies to arboviruses in the sera of children in Portuguese Guinea. Bulletin of the World Health Organization. 1967;37(1):101-8. Epub 1967/01/01. PubMed PMID: 5300043; PubMed Central PMCID: PMCPMC2554218.

20. Diallo M, Nabeth P, Ba K, Sall AA, Ba Y, Mondo M, et al. Mosquito vectors of the 1998–1999 outbreak of Rift Valley Fever and other arboviruses (Bagaza, Sanar, Wesselsbron and West Nile) in Mauritania and Senegal. 2005;19(2):119-26. doi: <https://doi.org/10.1111/j.0269-283X.2005.00564.x>.

21. Monlun E, Zeller H, Le Guenno B, Traoré-Lamizana M, Hervy JP, Adam F, et al. [Surveillance of the circulation of arbovirus of medical interest in the region of eastern Senegal]. Bulletin de la Societe de pathologie exotique (1990). 1993;86(1):21-8. Epub 1993/01/01. PubMed PMID: 8099299.

22. Renaudet J, Jan C, Ridet J, Adam C, Robin Y. [A serological survey of arboviruses in the human population of Senegal]. Bulletin de la Societe de pathologie exotique et de ses filiales. 1978;71(2):131-40. Epub 1978/03/01. PubMed PMID: 33772.

23. Mushi EZ, Binta MG, Raborokgwe M. Wesselsbron Disease Virus Associated with Abortions in Goats in Botswana. 1998;10(2):191-. doi: 10.1177/104063879801000216. PubMed PMID: 9576352.

24. Saluzzo J, Vincent T, Miller J, Veas F, Gonzalez J-P. Arbovirus Discovery in Central African Republic (1973-1993): Zika, Bozo, Bouboui, and More. Annals of Infectious Disease and Epidemiology. 2017;2.

25. van der Lugt JJ, Coetzer JA, Smit MM, Cilliers C. The diagnosis of Wesselsbron disease in a new-born lamb by immunohistochemical staining of viral antigen. The Onderstepoort journal of veterinary research. 1995;62(2):143-6. Epub 1995/06/01. PubMed PMID: 8600439.

26. EB Fokam LL, H Guzman, PA Amelia, VPK Titanji, RB Tesh, SC Weaver. Silent circulation of arboviruses in Cameroon. East African Medical Journal. 2010;87(6).

27. Barnard BJHV, Daniel Wynand. Antibodies against some viruses of domestic animals in southern African wild animals Onderstepoort Journal of Veterinary Research. 1997;64.

28. W.J. Van Der Riet FDSJ, Sayed, A.R., Barnard, B.J.H., Van Tonder, E.M., Crouse. Arthropod-borne virus zoonosis surveillance in the Cape Province: 1. prospective serological investigations for virus activity in the Beaufort West and Middelburg districts during 1981. 1985;56(1):25-9. doi: doi:10.10520/AJA00382809_2842.

29. Geser A, Henderson BE, Christensen S. A multipurpose serological survey in Kenya. 2. Results of arbovirus serological tests. Bulletin of the World Health Organization. 1970;43(4):539-52. Epub 1970/01/01. PubMed PMID: 5313066; PubMed Central PMCID: PMCPMC2427766.

30. Chippaux A, Chippaux-Hyppolite C, Monteny-Vandervorst N, Souloumiac-Deprez D. [Several yellow fever cases in an endemic area in Ivory Coast: serological and epidemiological evidence (author's transl)]. Medecine tropicale : revue du Corps de sante colonial. 1981;41(1):53-61. Epub 1981/01/01. PubMed PMID: 6268925.

31. Olaleye OD, Oladosu LA, Omilabu SA, Baba SS, Fagbami AH. Complement fixing antibodies against arboviruses in horses at Lagos, Nigeria. Revue d'elevage et de medecine veterinaire des pays tropicaux. 1989;42(3):321-5. Epub 1989/01/01. PubMed PMID: 2485538.

32. Fagbami AH, Fabiyi A. Arbovirus studies in two towns in western state of Nigeria. Tropical and geographical medicine. 1975;27(1):59-62. Epub 1975/03/01. PubMed PMID: 1138452.

33. Swanepoel R, Cruickshank JG. Arthropod borne viruses of medical importance in Rhodesia 1968-1973. The Central African journal of medicine. 1974;20(4):71-9. Epub 1974/04/01. PubMed PMID: 4842729.

34. Omilabu SA, Adejumo JO, Olaleye OD, Fagbami AH, Baba SS. Yellow fever haemagglutination-inhibiting, neutralising and IgM antibodies in vaccinated and unvaccinated residents of Ibadan, Nigeria. Comparative Immunology, Microbiology and Infectious Diseases. 1990;13(2):95-100. doi: <https://doi.org/10.1016/0147-9571(90)90521-T>.

35. Monath TP, Wilson DC, Lee VH, Stroh G, Kuteyi K, Smith EA. The 1970 yellow fever epidemic in Okwoga District, Benue Plateau State, Nigeria. I. Epidemiological observations. Bulletin of the World Health Organization. 1973;49(2):113-21. Epub 1973/01/01. PubMed PMID: 4545318; PubMed Central PMCID: PMCPMC2481124.

36. Guilherme JM, Gonella-Legall C, Legall F, Nakoume E, Vincent J. Seroprevalence of five arboviruses in Zebu cattle in the Central African Republic. Transactions of The Royal Society of Tropical Medicine and Hygiene. 1996;90(1):31-3. doi: 10.1016/S0035-9203(96)90468-X %J Transactions of The Royal Society of Tropical Medicine and Hygiene.

37. McIntosh BM, McGillivray GM, Dickinson DB, Malherbe H. ILLNESS CAUSED BY SINDBIS AND WEST NILE VIRUSES IN SOUTH AFRICA. South African medical journal = Suid-Afrikaanse tydskrif vir geneeskunde. 1964;38:291-4. Epub 1964/05/02. PubMed PMID: 14146460.

38. Weinbren MP, Kokernot RH, Smithburn KC. Strains of Sindbis-like virus isolated from culicine mosquitoes in the Union of South Africa. I. Isolation and properties. South African medical journal = Suid-Afrikaanse tydskrif vir geneeskunde. 1956;30(27):631-6. Epub 1956/07/07. PubMed PMID: 13351869.

39. Malherbe H, Strickland-Cholmley M, Jackson AL. Sindbis virus infection in man. Report of a case with recovery of virus from skin lesions. South African medical journal = Suid-Afrikaanse tydskrif vir geneeskunde. 1963;37:547-52. Epub 1963/05/25. PubMed PMID: 13932506.

40. Norder H, Lundström JO, Kozuch O, Magnius LO. Genetic Relatedness of Sindbis Virus Strains from Europe, Middle East, and Africa. Virology. 1996;222(2):440-5. doi: <https://doi.org/10.1006/viro.1996.0441>.

41. McIntosh BM, jupp PG. Infections in Sentinel Pigeons by Sindbis and West Nile Viruses in South Africa, With Observations on Culex (Culex) Univittatus (Diptera: Culicidae) Attracted to these Birds. Journal of Medical Entomology. 1979;16(3):234-9. doi: 10.1093/jmedent/16.3.234 %J Journal of Medical Entomology.

42. A.J. Jupp PG, Thompson, D.L., Cornel. Isolations of Middelburg virus from Aedes (Ochlerotatus) juppi McIntosh (Diptera: Culicidae) suggestive of a reservoir vector. 1987;50(2):393-7. doi: doi:10.10520/AJA00128789_4310.

43. NCBI Virus: Sindbis virus, taxid:11034 [Internet]. 2024. Available from: <https://www.ncbi.nlm.nih.gov/labs/virus/vssi/#/virus?SeqType_s=Nucleotide&Region_s=Africa&VirusLineage_ss=Sindbis%20virus,%20taxid:11034>.

44. Guarido MM, Fourie I, Meno K, Mendes A, Riddin MA, MacIntyre C, et al. Alphaviruses Detected in Mosquitoes in the North-Eastern Regions of South Africa, 2014 to 2018. 2023;15(2):414. PubMed PMID: doi:10.3390/v15020414.

45. Ochieng C, Lutomiah J, Makio A, Koka H, Chepkorir E, Yalwala S, et al. Mosquito-borne arbovirus surveillance at selected sites in diverse ecological zones of Kenya; 2007 – 2012. Virology Journal. 2013;10(1):140. doi: 10.1186/1743-422X-10-140.

46. Chiuya T, Masiga DK, Falzon LC, Bastos ADS, Fèvre EM, Villinger J. A survey of mosquito-borne and insect-specific viruses in hospitals and livestock markets in western Kenya. PloS one. 2021;16(5):e0252369. doi: 10.1371/journal.pone.0252369.

47. Mossel EC, Crabtree MB, Mutebi JP, Lutwama JJ, Borland EM, Powers AM, et al. Arboviruses Isolated From Mosquitoes Collected in Uganda, 2008-2012. J Med Entomol. 2017;54(5):1403-9. Epub 2017/09/07. doi: 10.1093/jme/tjx120. PubMed PMID: 28874015; PubMed Central PMCID: PMCPMC5968633.

48. Graff SL, Eibner GJ, Ochieng JR, Jones TC, Nsubuga AM, Lutwama JJ, et al. Detection of two alphaviruses: Middelburg virus and Sindbis virus from enzootic amplification cycles in southwestern Uganda. 2024;15. doi: 10.3389/fmicb.2024.1394661.

49. Sigei F, Nindo F, Mukunzi S, Ng’ang’a Z, Sang R. Evolutionary analyses of Sindbis virus strains isolated from mosquitoes in Kenya. Archives of Virology. 2018;163(9):2465-9. doi: 10.1007/s00705-018-3869-8.

50. Omondi D, Masiga DK, Ajamma YU, Fielding BC, Njoroge L, Villinger J. Unraveling Host-Vector-Arbovirus Interactions by Two-Gene High Resolution Melting Mosquito Bloodmeal Analysis in a Kenyan Wildlife-Livestock Interface. PloS one. 2015;10(7):e0134375. doi: 10.1371/journal.pone.0134375.

51. Jupp PG, Blackburn NK, Thompson DL, Meenehan GM. Sindbis and West Nile virus infections in the Witwatersrand-Pretoria region. South African medical journal = Suid-Afrikaanse tydskrif vir geneeskunde. 1986;70(4):218-20. Epub 1986/08/16. PubMed PMID: 3016922.

52. Adekolu-John EO, Fagbami AH. Arthropod-borne virus antibodies in sera of residents of Kainji Lake Basin, Nigeria 1980. Transactions of The Royal Society of Tropical Medicine and Hygiene. 1983;77(2):149-51. doi: 10.1016/0035-9203(83)90053-6 %J Transactions of The Royal Society of Tropical Medicine and Hygiene.

53. Johnson BK, Shockley P, Chanas AC, Squires EJ, Gardner P, Wallace C, et al. Arbovirus isolations from mosquitoes: Kano Plain, Kenya. Transactions of The Royal Society of Tropical Medicine and Hygiene. 1977;71(6):518-21. doi: 10.1016/0035-9203(77)90147-X %J Transactions of The Royal Society of Tropical Medicine and Hygiene.

54. KUNIHOLM MH, WOLFE ND, HUANG CY-H, MPOUDI-NGOLE E, TAMOUFE U, BURKE DS, et al. SEROPREVALENCE AND DISTRIBUTION OF FLAVIVIRIDAE, TOGAVIRIDAE, AND BUNYAVIRIDAE ARBOVIRAL INFECTIONS IN RURAL CAMEROONIAN ADULTS %J The American Journal of Tropical Medicine and Hygiene Am J Trop Med Hyg. 2006;74(6):1078-83. doi: 10.4269/ajtmh.2006.74.1078.

55. Woodall JP, Williams MC, Corbet PS, Haddow AJ. The Isolation of Sindbis Virus from the Mosquito Mansonia (Coquillettidia) Fuscopennata (Theobald) in Uganda. Annals of Tropical Medicine & Parasitology. 1964;58(4):383-9. doi: 10.1080/00034983.1964.11686260.

56. Attoui H, Sailleau C, Mohd Jaafar F, Belhouchet M, Biagini P, Cantaloube JF, et al. Complete nucleotide sequence of Middelburg virus, isolated from the spleen of a horse with severe clinical disease in Zimbabwe. 2007;88(11):3078-88. doi: <https://doi.org/10.1099/vir.0.83076-0>.

57. Kokernot RH, De Meillon B, Paterson HE, Heymann CS, Smithburn KC. Middelburg virus; a hitherto unknown agent isolated from Aedes mosquitoes during an epizootic in sheep in the eastern Cape Province. The South African journal of medical sciences. 1957;22(4):145-53. Epub 1957/12/01. PubMed PMID: 13529199.

58. Global Biodiversity Information Facility GOU. Aedes circumluteolus (Theobald, 1908) Occurrence Download. The Global Biodiversity Information Facility; 2024.

59. Tchouassi DP, Bastos AD, Sole CL, Diallo M, Lutomiah J, Mutisya J, et al. Population genetics of two key mosquito vectors of Rift Valley Fever virus reveals new insights into the changing disease outbreak patterns in Kenya. PLoS neglected tropical diseases. 2014;8(12):e3364. Epub 2014/12/05. doi: 10.1371/journal.pntd.0003364. PubMed PMID: 25474018; PubMed Central PMCID: PMCPMC4256213.

60. Global Biodiversity Information Facility GOU. Aedes mcintoshi Huang, 1985 Occurrence Download. The Global Biodiversity Information Facility; 2024.

61. Global Biodiversity Information Facility GOU. Culex univittatus Theobald, 1901 Occurrence Download. The Global Biodiversity Information Facility; 2024.

62. Global Biodiversity Information Facility GOU. Culex pipiens Linnaeus, 1758 Occurrence Download. The Global Biodiversity Information Facility; 2024.

63. Global Biodiversity Information Facility GOU. Mansonia africana (Theobald, 1901) Occurrence Download. The Global Biodiversity Information Facility; 2024.

64. Global Biodiversity Information Facility GOU. Aedes Meigen, 1818 Occurrence Download The Global Biodiversity Information Facility; 2024.

65. Global Biodiversity Information Facility GOU. Culex Linnaeus, 1758 Occurrence Download. The Global Biodiversity Information Facility; 2024.

66. Global Biodiversity Information Facility GOU. Mansonia Blanchard, 1902 Occurrence Download. The Global Biodiversity Information Facility; 2024.
