# Supplementary material for: Modeling the potential distribution of Wesselsbron, Sindbis, and Middelburg viruses and their vectors in Africa under future climatic and land-use changes
Source: PLoS Negl Trop Dis. 2026 Mar 4;20(3):e0014072. doi: 10.1371/journal.pntd.0014072 (PMC12970976; doi:10.1371/journal.pntd.0014072)
Supplement: S3 Table — (DOCX) [file pntd.0014072.s003.docx]

**S3 Table. Source datasets for ecological variables**

| **Variable*** | **Data set** | **Details*** | **Temporal extent** | **Resolution** | **Coordinate reference system** | **Data format** | **Reference** |
| --- | --- | --- | --- | --- | --- | --- | --- |
| **Current** | | | | | | | |
| Climate | WorldClim  Bioclimatic variables | 19 variables: **Bio1** = Annual Mean Temperature; **Bio2** = Mean Diurnal Range (Mean of monthly (max temp - min temp)); **Bio3** = Isothermality (Bio2/Bio7) (×100); **Bio4** = Temperature Seasonality (standard deviation ×100); **Bio5** = Max Temperature of Warmest Month; **Bio6** = Min Temperature of Coldest Month; **Bio7** = Temperature Annual Range (Bio5-Bio6); **Bio8** = Mean Temperature of Wettest Quarter; **Bio9** = Mean Temperature of Driest Quarter; **Bio10** = Mean Temperature of Warmest Quarter; **Bio11** = Mean Temperature of Coldest Quarter; **Bio12** = Annual Precipitation; **Bio13** = Precipitation of Wettest Month; **Bio14** = Precipitation of Driest Month; **Bio15** = Precipitation Seasonality (Coefficient of Variation); **Bio16** = Precipitation of Wettest Quarter; **Bio17** = Precipitation of Driest Quarter; **Bio18** = Precipitation of Warmest Quarter; **Bio19** = Precipitation of Coldest Quarter | 1970 - 2000 | 30 seconds (1 km at the equator) | WGS 84 lat/lon (EPSG 4326) | GeoTIFF | (Fick & Hijmans, 2017) |
| Normalized Difference Vegetation Index **(NDVI)** | MODIS/Terra Vegetation Indices Monthly L3 Global 1km SIN Grid V061 (MOD13A3.061) | Scaled integers divided by a factor of 10,000 to NDVI values -0.2 - 1 | 01/06/2015 to 30/06/2015 | 30 arc second or 1 km at the equator | WGS 84/Pseudo_Mercator(EPSG:3857) | GeoTIFF | (Didan, 2021) |
| Human population **(Hpop)** | Global Human Settlement Layer: Human Population (GHS-POP) | Population distribution by number of people per grid cell | 2015 | 30 arc second or 1 km at the equator | WGS84 | GeoTIFF | (Joint Research Centre - JRC - European Commission & Center for International Earth Science Information Network - CIESIN - Columbia University, 2021) |
| Built-Up areas **(Built)** | Global Human Settlement Layer: Built-Up Estimates (GHS-BUILT) | Proportion of the building footprint area within the total size of the grid cell | 2014 | 30 arc second or 1 km at the equator | WGS84 | GeoTIFF | (Joint Research Centre - JRC - European Commission & Center for International Earth Science Information Network - CIESIN - Columbia University, 2021) |
| Settlement model grid **(Smod)** | Global Human Settlement Layer: Settlement Model Grid or Degree of Urbanization (GHS-SMOD) | Grid cells assigned with 2-digit codes in the following classes: 30 = "Urban Centre" density of at least 1,500 inhabitants per square km; 23 = "Dense Urban Cluster" density of at least 1,500 inhabitants per square km; 22 = "Semi-dense Urban Cluster" density of at least 300 inhabitants per square km; 21 = "Suburban or peri-urban grid cells" all other cells of the Urban domain; 13 = "Rural cluster" density of at least 300 inhabitants per square km; 12 = "Low Density Rural grid cells" density of at least 50 inhabitants per square km; 11 = "Very low density rural grid cells" density of less than 50 inhabitants per square km; 10 = "Water grid cells" more than 0.5 share covered by permanent surface water | 2015 | 30 arc second or 1 km at the equator | WGS84 | GeoTIFF | (Joint Research Centre - JRC - European Commission & Center for International Earth Science Information Network - CIESIN - Columbia University, 2021) |
| Forested areas **(Forest)** | MCD12Q1_V61 Land Cover Type 1: Annual International Geosphere-Biosphere Programme (IGBP) of 17 classes | Forested areas extracted from the forest land cover classes: (1) Evergreen Needleleaf Forests; (2) Evergreen Broadleaf Forests; (3) Deciduous Needleleaf Forests; (4) Deciduous Broadleaf Forests; (5) Mixed Forests | 2015 | 500m | Sinusoidal | GeoTIFF | (Friedl, 2022) |
| Livestock density **(Livestock)** | Gridded Livestock of the World (GLW) Version 4 | The following species: cattle, sheep, goats, horses, pigs and chicken; absolute number of animals per pixel | 2015 | Approximately 10km (0.08333 degrees) | EPSG:4326 - WGS84 - Geographic Coordinate System (lat/long) | GeoTIFF | (NSAL, 2022) |
| Croplands **(Cropland)** | Global cropland expansion in the 21st century Cropland extent for 2012-2015. Pixel value: 0-100, percent of croplands per pixel | Cropland defined as land used for annual and perennial herbaceous crops for human consumption, forage (including hay), and Biofuel. Pixel value: 0-100, percent of croplands per pixel. | 2012-2015 | 0.025 × 0.025 degrees (~3 km × 3 km at the Equator) | EPSG:4326 - WGS 84 | GeoTIFF | (Potapov et al., 2022) |
| **Future** | | | | | | | |
| Climate | WorldClim Future Bioclimatic vraiables | CMIP6 climate projections; 19 Bioclim variables for GCMs IPSL CM6ALR and HadGEM3-GC3I-LL under SSP2-4.5 and SSP5-8.5 scenarios. Same 19 Bio climatic variables as current climate dataset | 2021 - 2040 | 30 seconds | WGS 84 lat/lon (EPSG 4326) | GeoTIFF | (Coupled Model Intercomparison Project Phase 6 (CMIP6), 2024) |
| Land use | GCAM-Demeter land use | Land-use files for GCMs IPSL CM6ALR and HadGEM3-GC3I-LL under SSP2-4.5 and SSP5-8.5 scenarios. Land-use types extracted from these classes: **Forest** = Classes of trees (1-8); **Cropland** = Classes of crops (15-30); **Urban** = Urban (31). Variables’ units are percent of grid area. | 2040 and 2060 | 0.05 degree |  | NetCDF | (Chen et al., 2020) |
| Human Population **(Hpop)** | Global gridded population data projected under shared socioeconomic pathways | Population distribution by number of people per grid cell under the SSPs projections | 2040 | 30 arc-seconds (approximately 1 km) | WGS84 projection | GeoTIFF | (Wang, Meng, & Long, 2022) |
| Livestock density **(Livestock)** | GLW 4: Gridded Livestock Density (Global - 2020 - 10 km) | head/pixel or birds/pixel | Reference year 2020 | 10km (0.08333 degrees) | EPSG:4326 - WGS84 | Float 64, GeoTiff | (NSAL, 2024) |

*Variable name as used in models and correlation matrix is marked in **bold font**

**References**

Chen, M., Vernon, C. R., Graham, N. T., Hejazi, M., Huang, M., Cheng, Y., & Calvin, K. (2020). Global land use for 2015–2100 at 0.05° resolution under diverse socioeconomic and climate scenarios. *Scientific Data, 7*(1), 320. doi:10.1038/s41597-020-00669-x

Coupled Model Intercomparison Project Phase 6 (CMIP6), W. (2024). *Future climate, 30 seconds spatial resolution*. Retrieved from: <https://www.worldclim.org/data/cmip6/cmip6_clim30s.html>

Didan, K. (2021). *MODIS/Terra Vegetation Indices Monthly L3 Global 1km SIN Grid V061*.

Fick, S. E., & Hijmans, R. J. (2017). WorldClim 2: new 1-km spatial resolution climate surfaces for global land areas. *37*(12), 4302-4315. doi:<https://doi.org/10.1002/joc.5086>

Friedl, M., Sulla-Menashe, D. (2022). *MODIS/Terra+Aqua Land Cover Type Yearly L3 Global 500m SIN Grid V061*. Retrieved from: <https://doi.org/10.5067/MODIS/MCD12Q1.061>

Joint Research Centre - JRC - European Commission, & Center for International Earth Science Information Network - CIESIN - Columbia University. (2021). *Global Human Settlement Layer: Population and Built-Up Estimates, and Degree of Urbanization Settlement Model Grid*. Retrieved from: <https://doi.org/10.7927/h4154f0w>

NSAL, F. a. A. O.-. (2022). *GLW 4: Gridded Livestock Density (Global - 2015 - 10 km)* [mapDigital]. Retrieved from: <https://data.apps.fao.org/catalog/iso/15f8c56c-5499-45d5-bd89-59ef6c026704>

NSAL, F. a. A. O.-. (2024). *GLW 4: Gridded Livestock Density (Global - 2020 - 10 km)* [mapDigital]. Retrieved from: <https://data.apps.fao.org/catalog/iso/9d1e149b-d63f-4213-978b-317a8eb42d02>

Potapov, P., Turubanova, S., Hansen, M. C., Tyukavina, A., Zalles, V., Khan, A., . . . Cortez, J. (2022). Global maps of cropland extent and change show accelerated cropland expansion in the twenty-first century. *Nature Food, 3*(1), 19-28. doi:10.1038/s43016-021-00429-z

Wang, X., Meng, X., & Long, Y. (2022). Projecting 1 km-grid population distributions from 2020 to 2100 globally under shared socioeconomic pathways. In: figshare.
