# Supplementary material for: Modeling the potential distribution of Wesselsbron, Sindbis, and Middelburg viruses and their vectors in Africa under future climatic and land-use changes
Source: PLoS Negl Trop Dis. 2026 Mar 4;20(3):e0014072. doi: 10.1371/journal.pntd.0014072 (PMC12970976; doi:10.1371/journal.pntd.0014072)
Supplement: S4 Table — (DOCX) [file pntd.0014072.s004.docx]

**S4 Table. Model settings and results per species**

| **Species** | ***Aedes cirumluteolus*** | ***Aedes mcintoshi*** | ***Culex pipiens*** | ***Culex univittatus*** | ***Mansonia africana*** | ***Wesselsbron virus*** | ***Sindbis virus*** | ***Middleburg virus*** |
| --- | --- | --- | --- | --- | --- | --- | --- | --- |
| Presence points | 121 | 89 | 241 | 172 | 157 | 154 | 109 | 49 |
| Variables selected from initial run in Maxent | Bio3, Bio6, Bio14, Bio18, Bio19, Built, Cropland, Forest, Hpop, Livestock | Bio3, Bio5, Bio6, Bio18, Bio19, Built, Cropland, Forest, Hpop, Livestock | Bio5, Bio7, Bio12, Bio17, Built, Cropland, Hpop, Livestock, NDVI | Bio6, Bio7, Bio12, Bio17, Built, Cropland, Forest, Hpop, Livestock | Bio3, Bio10, Bio11, Bio15, Bio17, Bio18, Built, Hpop, Livestock, NDVI | Aedes circumluteolus, Aedes mcintoshi, Bio3, Bio12, Bio13, Bio14, Bio15, Bio18, Built, Cropland, Forest, Hpop, Livestock | Culex pipiens, Culex univittatus, Bio5, Bio12, Built, Cropland, Hpop, Livestock, NDVI | Mansonia africana, Aedes mcintoshi, Bio1, Bio6, Bio9, Bio14, Bio15, Bio18, Built, Cropland, Forest, Hpop, Livestock, NDVI |
| **Candidate models** | | | | | | | | |
| Variable sets | 11 | 11 | 10 | 10 | 11 | 11 | 10 | 11 |
| Candidate models | 110 | 110 | 100 | 100 | 110 | 140 | 100 | 150 |
| Statistically significant based on pROC | 110 | 110 | 94 | 100 | 110 | 140 | 100 | 150 |
| Statistically significant + met OR + met AICc | 1 | 1 | 1 | 1 | 0 | 1 | 0 | 1 |
| Selected model parameters (Abbreviated as M_model's regularization multiplier_F_feature class combination_Set_set of variable combination) | M_5_F_lqpt_Set_2 | M_3_F_lqpth_Set_8 | M_5_F_lqp_Set_9 | M_3_F_lqpth_Set_2 | M_3_F_lq_Set_4 | M_5_F_lqpt_Set_11 | M_3_F_lqp_Set_7 | M_3_F_lqpt_Set_14 |
| Selected model Mean AUC ratio | 1.751 | 1.745 | 1.447 | 1.838 | 1.652 | 1.77 | 1.787 | 1.881 |
| **Final models** | | | | | | | | |
| Selected model's variables used for final model and projections | Bio3, Bio6, Bio14, Bio18, Bio19, Built, Cropland, Forest, Livestock | Bio5, Bio6, Bio18, Bio19, Built, Cropland, Forest, Hpop, Livestock | Bio5, Bio7, Bio17, Built, Cropland, Hpop, Livestock, NDVI | Bio6, Bio7, Bio12, Bio17, Built, Cropland, Forest, Livestock | Bio3, Bio10, Bio11, Bio15, Bio17, Bio18, Hpop, Livestock, NDVI | Aedes circumluteolus, Aedes mcintoshi, Bio3, Bio12, Bio13, Bio14, Bio15, Bio18, Built, Cropland, Forest, Hpop | Culex pipiens, Culex univittatus, Bio5, Bio12, Cropland, Hpop, Livestock, NDVI | Mansonia africana, Bio1, Bio6, Bio9, Bio14, Bio15, Bio18, Built, Cropland, Forest, Hpop, Livestock, NDVI |
| Final model Mean AUC ratio | 1.903 | 1.947 | 1.668606 | 1.935 | 1.986 | 1.921 | 1.984 | 1.889 |

**Key**

**Variable names in full: Bio1** = Annual Mean Temperature; Bio2 = Mean Diurnal Range (Mean of monthly (max temp - min temp)); **Bio3** = Isothermality (Bio2/Bio7) (×100); **Bio4** = Temperature Seasonality (standard deviation ×100); **Bio5** = Max Temperature of Warmest Month; **Bio6** = Min Temperature of Coldest Month; **Bio7** = Temperature Annual Range (Bio5-Bio6); **Bio8** = Mean Temperature of Wettest Quarter; **Bio9** = Mean Temperature of Driest Quarter; **Bio10** = Mean Temperature of Warmest Quarter; **Bio11** = Mean Temperature of Coldest Quarter; **Bio12** = Annual Precipitation; **Bio13** = Precipitation of Wettest Month; **Bio14** = Precipitation of Driest Month; **Bio15** = Precipitation Seasonality (Coefficient of Variation); **Bio16** = Precipitation of Wettest Quarter; **Bio17** = Precipitation of Driest Quarter; **Bio18** = Precipitation of Warmest Quarter; **Bio19** = Precipitation of Coldest Quarter; **NDVI =** Normalized Difference Vegetation Index; **Hpop** = Human population; **Built** = Built-Up areas; **Smod** = Settlement model grid; **Forest** = Forested areas; **Livestock** = Livestock density; **Cropland** = Croplands

l = linear; p = product; q = quadratic; h = hinge; t = threshold; AICc = Akaike Information criterion corrected for small sample sizes; pROC = partial Receiver Operating Curve; OR = Omission Rate below error of 5%; AUC = Area Under the Curve
