# Supplementary material for: Modeling the potential distribution of Wesselsbron, Sindbis, and Middelburg viruses and their vectors in Africa under future climatic and land-use changes
Source: PLoS Negl Trop Dis. 2026 Mar 4;20(3):e0014072. doi: 10.1371/journal.pntd.0014072 (PMC12970976; doi:10.1371/journal.pntd.0014072)
Supplement: S5 Table — (DOCX) [file pntd.0014072.s005.docx]

**S5 Table. Area of mosquito species habitat suitability and percent change per scenario**

| **Species** | **Class** | **Area for Current in 10,000 km^2^** | **Area for SSP245 in 10,000 km^2^** | **Percent Difference SSP245** | **Area for SSP585 in 10,000 km^2^** | **Percent Difference**  **585** |
| --- | --- | --- | --- | --- | --- | --- |
| ***Aedes circumluteolus*** | 0-0.3 | 2,359.86 | 3,360.92 | 42.42 | 3,377.06 | 43.10 |
|  | 0.4-0.6 | 568.46 | 351.23 | -38.21 | 341.72 | -39.89 |
|  | 0.7-1.0 | 176.48 | 89.19 | -49.46 | 82.56 | -53.22 |
| ***Aedes mcintoshi*** | 0-0.3 | 2,346.46 | 3,097.89 | 32.02 | 3,103.70 | 32.27 |
|  | 0.4-0.6 | 656.72 | 629.94 | -4.08 | 627.79 | -4.40 |
|  | 0.7-1.0 | 101.43 | 69.79 | -31.20 | 66.13 | -34.80 |
| ***Culex pipiens*** | 0-0.3 | 2,172.11 | 3,023.92 | 39.22 | 3,181.36 | 46.46 |
|  | 0.4-0.6 | 910.40 | 767.54 | -15.69 | 610.18 | -32.98 |
|  | 0.7-1.0 | 24.66 | 6.16 | -75.01 | 6.08 | -75.35 |
| ***Culex univittatus*** | 0-0.3 | 2,937.47 | 3,682.00 | 25.35 | 3,519.13 | 19.80 |
|  | 0.4-0.6 | 135.32 | 117.50 | -13.17 | 279.20 | 106.33 |
|  | 0.7-1.0 | 31.40 | 1.84 | -94.15 | 3.01 | -90.40 |
| ***Mansonia africana*** | 0-0.3 | 2,733.22 | 3,381.47 | 23.72 | 3,359.74 | 22.92 |
|  | 0.4-0.6 | 333.96 | 377.58 | 13.06 | 389.52 | 16.64 |
|  | 0.7-1.0 | 40.00 | 38.74 | -3.15 | 48.53 | 21.32 |
