# Supplementary figures and images for: Modeling the potential distribution of Wesselsbron, Sindbis, and Middelburg viruses and their vectors in Africa under future climatic and land-use changes
Source: PLoS Negl Trop Dis. 2026 Mar 4;20(3):e0014072. doi: 10.1371/journal.pntd.0014072 (PMC12970976; doi:10.1371/journal.pntd.0014072)

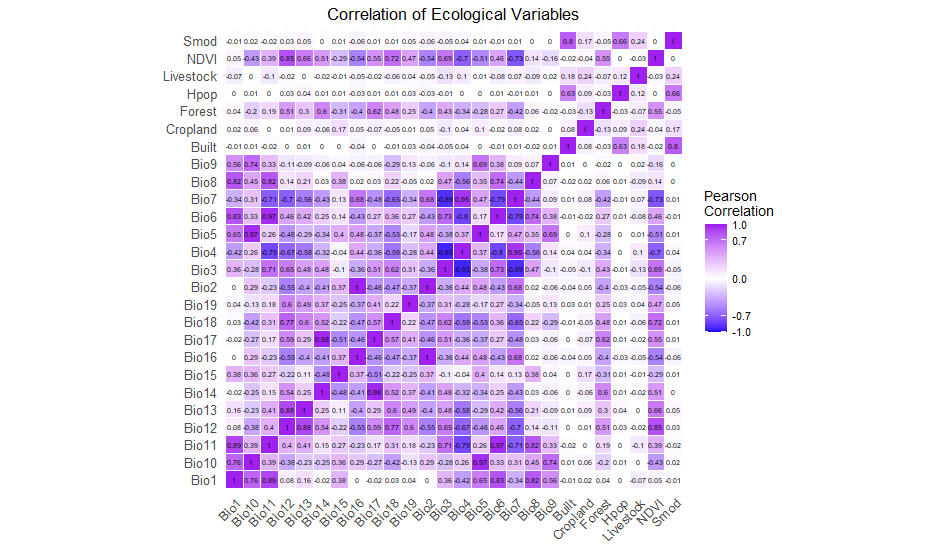

Supplement: S1 Fig — (PNG) [file pntd.0014072.s006.png]
